# Multidisciplinary Preclinical Investigations on Ferrocenyl, Ruthenocenyl, and Benzyl Derivatives of Niridazole as New Drug Candidates against Schistosomiasis

**DOI:** 10.1002/cmdc.202500603

**Published:** 2025-11-04

**Authors:** Tanja Karpstein, Shuai Zhong, Sarah Keller, Philipp Späne, Cécile Häberli, Gordana Panic, Olivier Blacque, Alex Odermatt, Kevin Cariou, Gilles Gasser, Jennifer Keiser

**Affiliations:** ^1^ Department of Medical Parasitology and Infection Biology Swiss Tropical and Public Health Institute Kreuzstrasse 2 CH‐4123 Allschwil Switzerland; ^2^ University of Basel CH‐4003 Basel Switzerland; ^3^ Chimie ParisTech PSL University CNRS Laboratory for Inorganic Chemical Biology Institute of Chemistry for Life and Health Sciences 75005 Paris France; ^4^ Division of Molecular and Systems Toxicology Department of Pharmaceutical Sciences University of Basel Klingelbergstrasse 50 4056 Basel Switzerland; ^5^ Department of Chemistry University of Zurich 8001 Zurich Switzerland; ^6^ Swiss Centre for Applied Human Toxicology and Department of Pharmaceutical Sciences University of Basel Missionsstrasse 64 4055 Basel Switzerland

**Keywords:** bioorganometallic chemistry, drug discovery, ferrocene, niridazole, schistosomiasis

## Abstract

Schistosomiasis affects hundreds of millions of people worldwide, yet its chemotherapeutic treatment is based on the only drug available‐praziquantel (PZQ). The development of alternative treatment options is urgent, not only due to the threat of drug resistance, but also because the drawbacks of PZQ, such as its inactivity against juvenile stages, contribute to its incomplete cure rates, thus requiring repeated treatment. This study presents the design, synthesis, characterization, and biological evaluation of 10 novel organometallic derivatives of the old schistosomicide, niridazole. The in vitro characterization of the derivatives on different life stages of *Schistosoma mansoni* showed that the activity profile of niridazole could be modified and extended. One ferrocenoyl derivative showed promising activity against all life cycle stages of *S. mansoni*. Two ferrocenyl and one ruthenocenyl derivatives also displayed higher potency against adult schistosomes than niridazole. In conclusion, valuable information could be gained on the structure–activity relationship of the different organometallic modifications, which could be used to design a second generation of derivatives with further improved activity profiles.

## Graphical abstract

Novel organometallic derivatives of niridazole are designed, synthesized, and tested against *Schistosoma mansoni*.

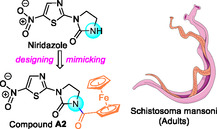

## 1 Introduction

Schistosomiasis is both an acute and chronic parasitic disease in tropical and subtropical regions caused by blood‐dwelling trematodes of the genus *Schistosoma.* It is one of the most prevalent neglected tropical diseases (NTDs) in the world, with 800 million people at risk of being infected by one of the six *Schistosoma* species infecting humans. *Schistosoma haematobium*, *Schistosoma japonicum*, and *Schistosoma mansoni* are the clinically most relevant schistosome species.^[^ ^1^, ^2^ ^–^ ^3^ ^]^ Depending on the infecting species, two different forms of schistosomiasis exist. *S. haematobium* infection leads to urogenital schistosomiasis, while *S. mansoni* and *S. japonicum* infections cause the intestinal form of the disease. Chronic infections can have severe consequences such as fibrosis, renal failure, bladder cancer, or infertility.^[^ ^2^ ^]^ Schistosomiasis causes an estimated burden of 1.7–4.5 million disability‐adjusted life years through impeding morbidities such as anemia, malnutrition, and impaired development, as well as higher vulnerability to co‐infection with other NTDs.^[^ ^4^ ^,^ ^5^ ^]^

Treatment and control are based on praziquantel (PZQ), the only drug available.^[^ ^6^ ^]^ Mass drug administration programs aim to reduce the morbidity with biannual or annual treatment with PZQ. Due to this widespread use for decades, the risk of resistance emergence and consequently treatment failure is high.^[^ ^7^ ^]^ Although PZQ has a good safety profile and is active against all medically important *Schistosoma* species, single‐dose administration is not curative, primarily due to its lacking efficacy against juvenile worms.^[^ ^8^ ^,^ ^9^ ^]^ Therefore, the need for novel treatment options is urgent.

As is typical for drug discovery in NTDs, a lack of resources and insufficient drug target information hinder research. Unlike some other NTDs, drug discovery for schistosomiasis is mainly driven by academia. Through support by private−public partnerships, global alliances, and philanthropic institutions, an enhanced research landscape has emerged over the past decade. To target the elimination of schistosomiasis, the development of new drugs that are effective at a single dose and exhibit broad activity against all life stages of the parasites will be essential. To be compatible for large‐scale administration, further requirements are oral activity, stability under warm and humid conditions, and low production cost.^[^ ^8^ ^,^ ^10^ ^]^

Since de novo drug discovery is time‐intensive and expensive, approaches like drug repurposing and structure‐based optimization of already existing drugs are widely used to find novel antischistosomal drug candidates.^[^ ^11^, ^12^, ^13^ ^–^ ^14^ ^]^ As proven by the success of the antimalarial drug candidate ferroquine, a ferrocenyl derivative of chloroquine that entered the clinical phase, derivatization of a known organic drug with organometallic moieties can help to improve its activity and safety profiles.^[^ ^15^, ^16^, ^17^ ^–^ ^18^ ^]^ Introduction of an organometallic moiety can generate new and unique metal‐specific modes of action, leading to optimized derivatives with enhanced activities compared to their parent compounds.^[^ ^19^ ^,^ ^20^ ^]^

The old schistosomiasis drug niridazole could be a good candidate for organometallic derivatization, since it is one of the most effective schistosomicides. Niridazole, a nitrothiazole derivative, is active against *S. mansoni*, *S. haematobium* and *S. japonicum* infections and is also very effective in the treatment of guinea worm infection. The recommended treatment regimen is an oral dose of 25–30 mg kg^–1^ over a period of five to ten days.^[^ ^21^, ^22^, ^23^ ^–^ ^24^ ^]^ However, apart from the inconvenient multiple dosing scheme, several factors have led to niridazole's characterization as clinically not acceptable. Rare but severe occurrences of drug‐induced central nervous system toxicity with adverse events including convulsions and acute psychoses, as well as immunosuppressive, mutagenic, and carcinogenic effects of the drug finally, caused its withdrawal from the market.^[^ ^22^ ^,^ ^25^, ^26^, ^27^ ^–^ ^28^ ^]^ Severe side effects occurred particularly in patients with hepatosplenic schistosomiasis.^[^ ^29^, ^30^ ^–^ ^31^ ^]^

**Figure 1:**
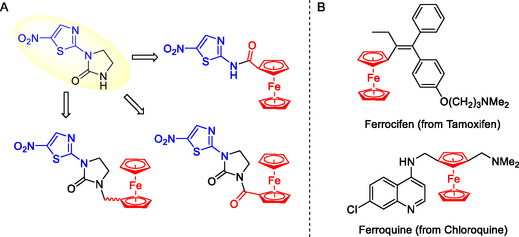
A) Chemical structures of Niridazole and its ferrocenyl derivatives, B) Ferrocene‐containing drug candidates.

The derivatization of existing drugs with metallocenes started to boom in the 1990s when ferroquine and ferrocifen were designed and developed by the groups of Brocard and Jaouen, respectively.^[^ ^15^ ^,^ ^32^ ^,^ ^33^ ^]^ In both cases, the incorporation of the ferrocenyl moiety into the organic drug led to superior activities compared to their parent drugs. (**Figure** **1**B) The additional activities were shown to be caused, among others, by the generation of reactive oxygen species via the reversible ferrocene/ferrocenium redox couple.^[^ ^34^ ^]^ In the following year, many research groups around the world have been interested in derivatizing known patent drugs with metallocenes to create compounds with novel properties and additional modes of action.^[^ ^35^ ^]^

Here we describe a simple and efficient approach of organometallic derivatization, which has already been successfully applied to other anthelmintic drugs like albendazole, mefloquine, oxamniquine, and monepantel.^[^ ^12^ ^,^ ^35^, ^36^, ^37^, ^38^, ^39^ ^–^ ^40^ ^]^ Our aim is to improve the activity profile of niridazole and ultimately to overcome its toxic side effects. In this work, we present the synthesis and complete characterization of ten new (organometallic) niridazole derivatives and their in‐depth biological activity on different life stages of *S. mansoni*.

## 2 Results

### 2.1 Synthesis and Characterization

**Scheme 1:**
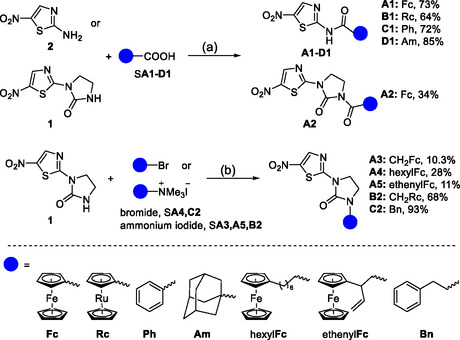
Synthesis of target compounds **A1‐A5, B1‐B2, C1‐C2,** **D1**. Reagents and conditions: a) DIPEA, HATU or TBTU, DMF, RT, overnight; b) K_2_CO_3_, MeCN, reflux, overnight.

Thiazoles are five‐membered heteroaromatic rings containing a nitrogen and a sulfur at the 1,2 or the 1,3 positions. Niridazole contains a 1,3‐thiazole ring with a nitro at the 5‐position and a cyclic urea (imidazolidinone) at the 2‐position (Figure 1A). Herein, we use a straightforward strategy by replacing or modifying the imidazolidinones in niridazole with a ferrocene moiety (Fc) to produce **A1** **–A5**. The aim was to improve the bioactivity of niridazole (**Scheme** **1**). The remarkable physico‐chemical properties of ferrocene (e.g., nontoxic, stable, robust, lipophilic, and redox reversible) made it one of the most popular organometallic moieties in medicinal organometallic chemistry. In order to understand the role of the ferrocenyl moiety in the activity of the new compounds prepared, we decided to use several controls. To be able to identify the effects of the metallocene moieties, we synthesized the ruthenocenyl derivatives (Rc) **B1** **–B2**, as well as phenyl (Ph) **C1**, adamantyl (Am) **D1**, and benzyl (Bn) **C** **2** analogs. Rc has a very similar geometry and steric demand to Fc. However, Rc displays very different redox properties compared to Fc.^[^ ^41^ ^]^ The phenyl and benzyl groups can be used as controls to assess the impact of adding an aromatic moiety on the drug efficacy. Adamantane is a tricyclic cage compound that is not aromatic. However, it emulates the 3D barrel shape of the metallocenes. The incorporation of adamantane fragments in pharmaceuticals was shown to improve the lipophilicity and stability of drugs.^[^ ^42^ ^,^ ^43^ ^]^

Two different reaction conditions were applied to synthesize the niridazole analogs, depending on the link between the organometallic group and the niridazole core. The amide niridazole derivatives (**A1–D1**, **A2**) were obtained by employing the known activating agents HATU or TBTU with yields ranging from 64% to 85%. Compounds **A3–A5**, **B2**, and **C2** were prepared by *N*‐alkylation of niridazole with various bromide or trimethylammonium iodide derivatives. All newly synthesized compounds were unambiguously characterized by ^1^H and ^13^C NMR spectroscopy and high‐resolution mass spectrometry (HR‐MS), while their purities were confirmed by elemental analysis. Details on the synthesis and characterization of the complexes can be found in the Supporting Information.

All compounds included were then categorized into two different series. Compounds **A2–A5**, **B2**, and **C2** belonged to Series I, where an aromatic or organometallic moiety was appended to the imidazolidone part of niridazole by acylation or alkylation of the N—H bond in the imidazolidinone ring of niridazole. In Series II, that is compounds **A1–D** **1**, the imidazolidone part of niridazole was replaced with either an aliphatic, aromatic, or organometallic unit. Another difference is that the nitrogen attached to the thiazole ring is a secondary amine in Series II, whereas in niridazole and in Series I, this nitrogen is trisubstituted.

### 2.2 X‐Ray Crystallography

**Figure 2:**
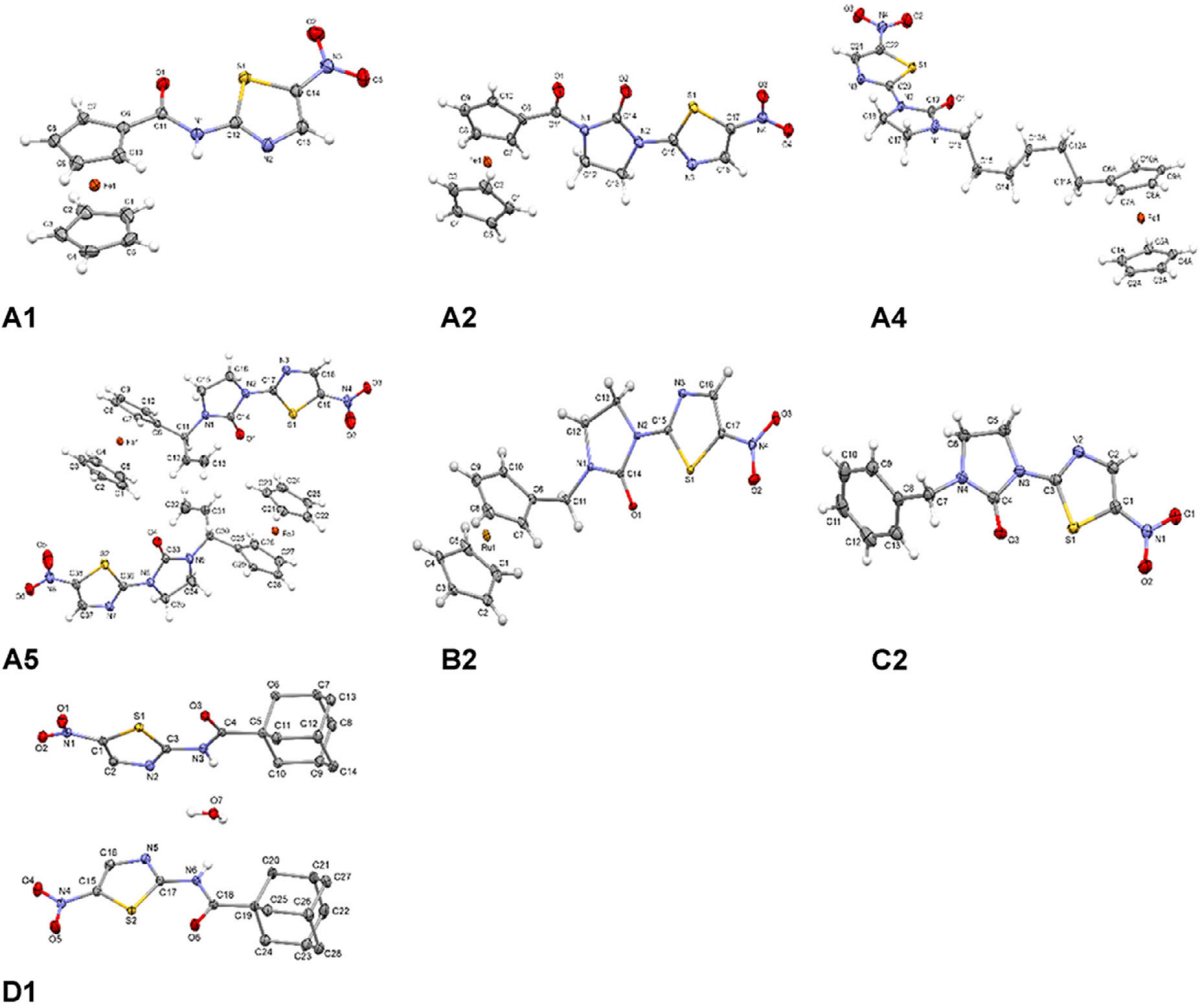
POV‐ray representation of compounds **A1‐A2**, **A4‐A5**, **B2**, **C2**, and **D1**. The most relevant crystallographic data is collected in ESI, see Table S4–S7, Supporting Information.

Single‐crystal structure determinations were carried out for seven niridazole derivatives synthesized in this study (**A1‐A2**, **A4‐A5**, **B2**, **C2**, and **D1**; **Figure** **2**). In all cases, the crystal structures confirmed the identity of the complexes. Among some notable features, the crystal structure of **A2** was refined as a two‐component nonmerohedral twin [scale: 0.1163(14) /0.8837(14)]. The minor component was rotated by 179.96° around [0.0 0.0 1.0] in the reciprocal space or around [0.38 0.00 0.93] in the direct space. Compound **A** **4** has special features that the ferrocene (except the metal center) and three CH_2_ groups of the C6 chain are disordered over two sets of positions (named A and B) with site‐occupancy factors of 0.494(2) and 0.506(2). Also, we found that the crystal structure of **A5** has two crystallographically independent molecules in the asymmetric unit. The crystal structure of **B2**, the solvent molecule of acetone, is disordered over two sets of positions with a site‐occupancy factor of 0.5. Details on the acquisition of the structures can be found in the Supporting Information.

### 2.3 Stability of Compounds in DMSO

Stability is a key factor that needs to be investigated in medicinal chemistry, particularly with metal complexes.^[^ ^44^ ^]^ We randomly chose four niridazole derivatives that we dissolved in DMSO‐*d*_6_ (**A3**, **B1**, **C2**, and **D1**). We then monitored by ^1^H NMR spectroscopy any change in the spectra after 0, 6, 24, 30, and 48 h at room temperature. No shifted peaks or new peaks were found for all the tested compounds over 2 days, showing that they are stable in DMSO‐*d*_6_. The NMR spectra of these studies can be found in the Supporting Information (Figure S32–S35).

### 2.4 In Vitro Drug Efficacy Tests

The activity profile of the new organometallic compounds on different species and life stages was determined in in vitro tests.

**Figure 3:**
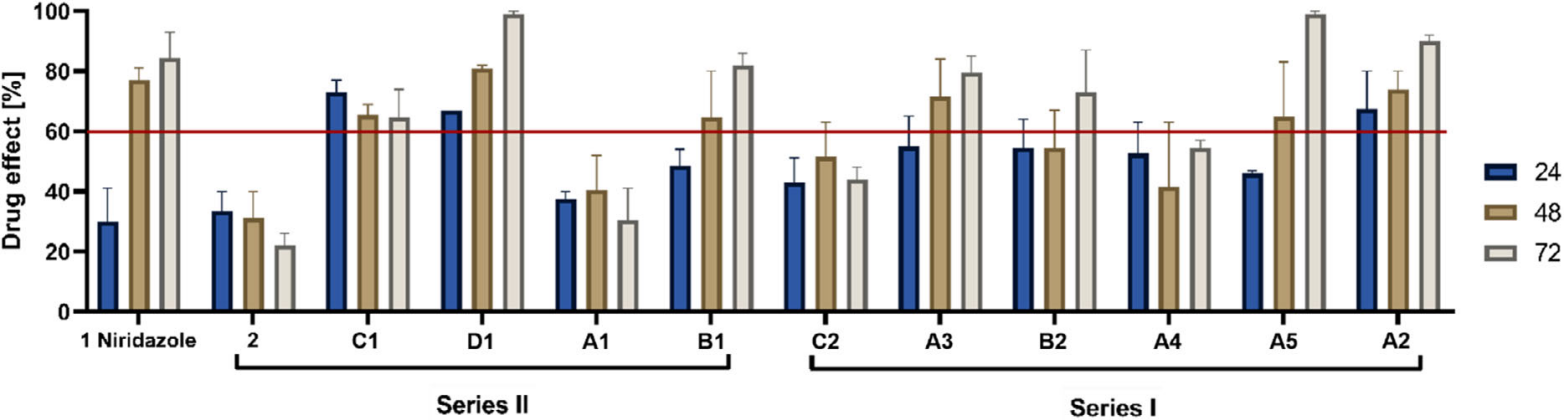
In vitro activity at 50 µM of the 12 compounds on adult *S. mansoni*. Adult schistosomes were incubated in culture media for 72 h at 37 °C and 5% CO_2_ with either compound (50 µM) or a DMSO (0.5%) control. The viability of the worms was microscopically evaluated and scored (0—3, in 0.5 steps) every 24 h. Results are from two independent experiments, each performed in duplicate. Drug activities were normalized to the DMSO controls. Data represent mean ± SD in percentage of DMSO control. A compound was classified as active when the drug effect was ≥60% (indicated as a red line).

The synthesized organometallic derivatives were tested against *S. mansoni* newly transformed schistosomula (NTS), juvenile, and adult worms. **Figure** **3** shows the drug effects at 50 µM of the test compounds on adult *S. mansoni* (mean ± SD). The red line indicates our selected threshold of 60% drug effect for a compound to be considered active. Eight compounds were active at 50 µM against adult *S. mansoni* after 72 h of drug incubation, with activities ranging from 64–99%. Compounds **A5** and **D1** were able to kill nearly all worms (99% average drug effect) after 72 h of incubation at 50 µM. The parent compound niridazole reached a drug effect of 83% after 72 h of incubation. Compounds **A** **3**, **B1**, **B2**, and **C1** were less active than niridazole, while the compounds **A2**, **A5**, and **D1** were more active after 72 h. Most of the active compounds showed increasing activity over time (lowest drug effect after 24 h and highest after 72 h of incubation).

When further tested at 10 µM on adult *S. mansoni*, four of the seven active compounds still showed mean drug effects above 60% after 72 h of incubation (see Table S1, Supporting Information). The compounds with the methylene‐ferrocene (compound **A3**) and ruthenocene (compound **B2**) moieties exhibited very similar drug effects at 10 µM. Compound **A5** with the additional ethenyl attachment on the ferrocenyl moiety was the only one that was still able to kill the adult schistosomes at 10 µM after 72 h. Compound **C1**, which contains a phenyl group, was the only compound to show a maximum drug effect already after 48 h of drug incubation.

**Figure 4:**
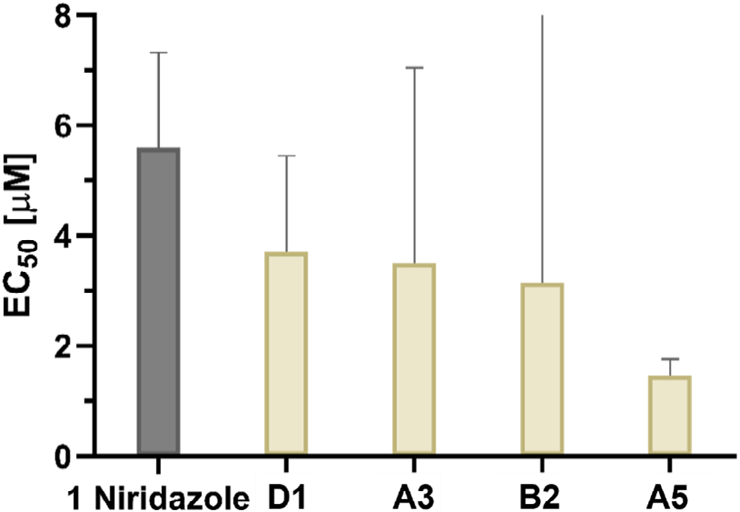
In vitro potency after 72 h of the four active derivatives compared to niridazole. The in vitro potency results on adult *S. mansoni* are presented as EC_50_ [µM] with their 95% confidence intervals (CI) as error bars after 72 h of drug exposure at 37 °C and 5% CO_2_. Data are from two independent experiments, each performed in duplicates. The 95% CI error bar for compound **B2** is clipped; the upper limit of the 95% CI is 16.8 µM.

The EC_50_ values for niridazole and the organometallic derivatives compounds **A3**, **A5**, **B2**, and **D1** on adult *S. mansoni* were subsequently determined using serial dilution tests. The EC_50_ results of the 72 h drug exposure are depicted in **Figure** **4** (and Table S1, Supporting Information). Niridazole showed an EC_50_ value of 5.6 µM. All the evaluated derivatives were more potent than niridazole against adult *S. mansoni*. Compound **A5** with the additional ethenyl group at the ferrocenyl moiety showed the highest potency with an EC_50_ value of 1.5 µM after 72 h, translating to a 3.8‐fold higher activity than niridazole. The results of the structurally similar compounds **A3** (ferrocenyl moiety) and **B2** (ruthenocenyl moiety) were similar, with 1.6 (compound **B2**) and 1.8 (compound **A3**) times higher potencies than niridazole. The ruthenocenyl‐containing compound **B2** revealed an EC_50_ value of 3.2 µM compared to its ferrocenyl‐containing counterpart compound **A3**, which showed an EC_50_ of 3.5 µM after 72 h. Compound **D** **1** showed a 1.5‐fold higher potency than niridazole with an EC_50_ of 3.7 µM at 72 h post‐incubation.

When comparing the potencies over time (**Table** **1**), it is visible that all compounds, including niridazole, exert their effect only after 48 h of incubation, except for compound **D1**, which revealed an EC_50_ value below 10 µM at 24 h post‐incubation. The EC_50_ values for compound **A5** and niridazole after 24 h of incubation were above the highest tested concentration of 50 µM.

**Table 1:** In vitro potency over time of the five most active compounds. The in vitro potency of the compounds on adult S. mansoni is presented as EC_50_ [µM] after 24, 48, and 72 h of drug exposure at 37 °C and 5% CO_2_. Additionally, the lower and upper 95% confidence intervals (CI) as well as the R^2^ values are shown for the calculated EC_50_ values. Based on two independent experiments, each performed in duplicate.

Structure	Compound	Parameter	24 h	48 h	72 h	Fold activity of niridazole
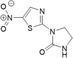	Niridazole/1	EC_50_ [µM]	>50	7.8	5.6	–
		95% CI lower	NA	4.4	4.1	
		95% CI upper	NA	12.8	7.3	
		R^2^	0.6	0.8	0.9	
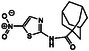	D1	EC_50_ [µM]	9.8	4.2	3.7	1.5
		95% CI lower	5.5	1.7	2.4	
		95% CI upper	19.3	8.3	5.5	
		R^2^	0.9	0.8	0.9	
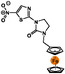	A3	EC_50_ [µM]	50.5	9.7	3.5	1.6
		95% CI lower	NA	3.1	1.5	
		95% CI upper	NA	51.1	7.1	
		R^2^	0.2	0.7	0.8	
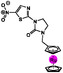	B2	EC_50_ [µM]	38.1	NA	3.2	1.8
		95% CI lower	13.5	NA	0	
		95% CI upper	3266	NA	16.8	
		R^2^	0.6	NA	0.5	
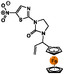	A5	EC_50_ [µM]	>50	5.3	1.5	3.8
		95% CI lower	12.5	NA	1.2	
		95% CI upper	NA	NA	1.8	
		R^2^	0.2	0.5	1.0	

In parallel, the compounds were tested on NTS and juvenile *S. mansoni*. **Table** **2** shows the EC_50_ values after 72 h of exposure to all compounds on all stages. The only compound from series I that was active against all life stages of *S. mansoni* is compound **A2**, which is also the compound with the strongest activity against NTS, with an EC_50_ value of 2.0 µM. From series II, compounds **C1** and **D1** also showed activity against NTS, but they were less potent with EC_50_ values of 3.1 µM and 7.9 µM, respectively.

**Table 2:** Stage specificity. Summary of all EC_50_ values [µM] after 72 h of incubation at 37 °C and 5% CO_2_ on *S. mansoni* on all tested life stages. nd= not determined.

Compound	EC_50_ [µM] values after 72 h		
	Adults	Juvenile	NTS
Niridazole	5.6	1.8	>50
2	>50	nd	>50
A1	>50	nd	>50
B1	6.9	>50	>50
C1	11.6	>50	3.1
D1	3.7	29.25	7.9
A2	8.2	6.35	2.0
A3	3.5	5.7	>50
A4	>50	nd	>50
A5	1.5	12.2	>50
B2	3.2	8.0	>50
C2	>50	nd	>50

Compounds **A2**, **A3**, **A5**, **B2**, and niridazole, which showed the strongest effect with an EC_50_ of 1.8 µM, were also active against *S. mansoni* juveniles. None of the compounds from series II were active against juvenile schistosomes. All of the series I compounds that were potent against the adult stage were also active against the juveniles. The derivatives with basic ferrocenyl‐ and ruthenocenyl‐groups (compounds **A3** and **B2**), which showed excellent activity against adult *S. mansoni*, were also potent against juveniles, albeit somewhat less pronounced. No drug effect was observed at the highest tested concentration of 50 µM against any of the *S. mansoni* life stages for compounds **2**, **A1**, **A4**, and **C2**.

### 2.5 Cytotoxicity Testing

The safety profile of niridazole and its organometallic derivatives was evaluated using two different cell lines. The XTT mitochondrial activity assay reflecting cell viability was performed in the hepatocyte‐derived carcinoma cell line HUH7 and in the human neuroblastoma cell line SH‐SY5Y at 20 µM for 24 h. The SYTOX cell viability assay was subsequently performed on the SH‐SY5Y cell line, once the cell line was identified to be more sensitive to the treatment conditions.

**Figure 5:**
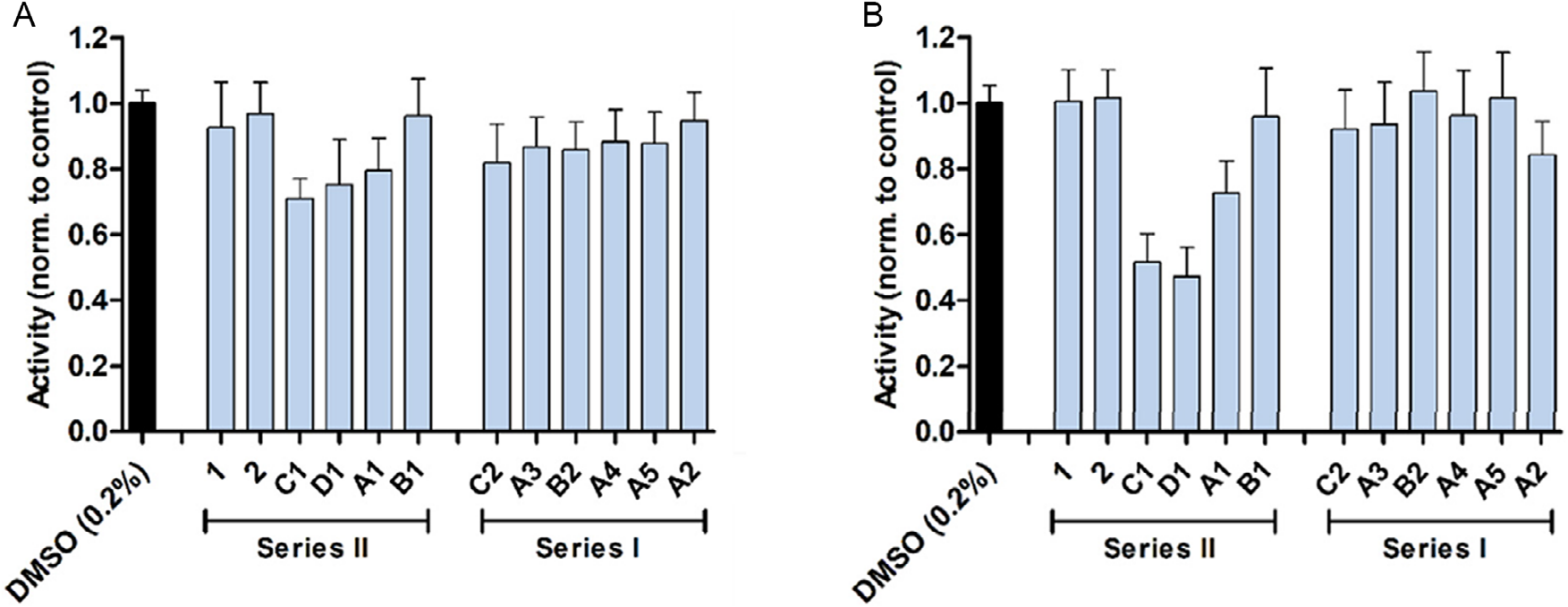
XTT assay. Results of the XTT activity/viability assay are shown in A for HUH7 and B for SHSY5Y cells. Cells were treated with either compound (20 µM) or DMSO control (0.2%) for 24 h at 37 °C and 5% CO_2_ in serum‐free media. Results were normalized to the DMSO control. Data represent mean ± SD from three independent experiments, each performed in quintuplicate.

In **Figure** **5**, the normalized activities measured by the XTT mitochondrial activity/cell viability assay are indicated for HUH7 (A) and SH‐SY5Y cells (B). Compounds **A1**, **C1**, and **D1** induced a loss of activity compared to the DMSO control (0.2%) in both cell lines. The reduction in activity was more prominent in SH‐SY5Y neuroblastoma cells compared to the HUH7 hepatocarcinoma cells. Compounds **C1** reduced the signal by 48% (mean) and compound **D1** by 53% (mean), while compound **A** **1** reduced the signal by 27% in the SH‐SY5Y.

**Figure 6:**
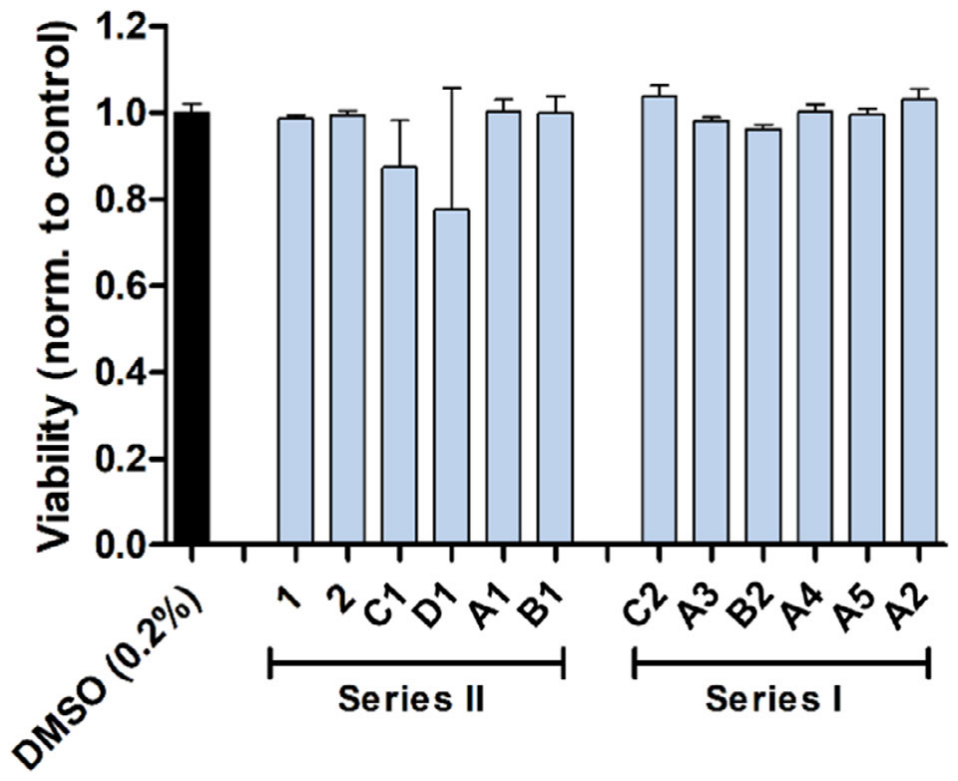
SYTOX cell viability assay.

The SYTOX cell viability test was subsequently performed on the more sensitive SH‐SY5Y cell line (**Figure** **6**). Treatment with compound **C1** and **D1** resulted in a reduced signal, while compound **A1** did not alter SH‐SY5Y cell viability. Compound **C1** reduced cell viability by 13% (mean) and compound **D1** by 23% (mean) after 24 h of treatment at 20 µM.

The SYTOX cell viability assay was performed in SHSY5Y cells. Cells were treated with either compound (20 µM) or DMSO control (0.2%) for 24 h at 37 °C and 5% CO_2_ in serum‐free charcoal‐treated medium. Results were normalized to the DMSO control. Mean ± SD was calculated from three independent experiments, each performed in quadruplicate.

## 3 Discussion

The declared aim to eliminate schistosomiasis as a public health problem by 2030^[^ ^3^ ^]^ requires the development of novel schistosomicides active against all life stages and all *Schistosoma* species. Additionally, single‐dose administration, oral formulation, and low cost are essential to make the drug suitable for broad distribution in mass drug administration programs in tropical regions.^[^ ^8^ ^,^ ^10^ ^]^ The given resource limitations and the pressing need for alternative treatment require adapted drug discovery processes like drug repurposing and the structure‐based optimization of already existing drugs.^[^ ^11^ ^]^ In this study, we could show that the old schistosomicide niridazole can be optimized through organometallic derivatization. We found a promising derivative (compound **A2**) with good activity against all developmental stages of *S. mansoni*, as well as several derivatives (compounds **A3**, **A5**, **B2**, and **D1**) with enhanced potency against adult schistosomes. Overall, compound **A2** could be an ideal drug candidate with its broad activity against NTS, juvenile and adult forms of *S. mansoni*, and its safe cytotoxicity profile.

The evaluation of the drug activity on the three different life stages of *S. mansoni* showed that, as expected for an old schistosomicide, niridazole was active against adult *S. mansoni* (EC_50_ value of 5.6 µM) and even not only more potent, but also the most potent of all tested compounds against juvenile schistosomes (EC_50_ value of 1.8 µM). The early developing form, the NTS stage, was not impaired by niridazole when tested at 50 µM.

Drug activity on adult schistosomes was observed for all compounds from series I, except for the benzyl‐group containing compounds **C2** and compound **A4** with the ferrocenyl‐group attached via a longer (*n‐*hexyl) alkyl chain. From a structure–activity relationship (SAR) perspective, this indicates that the organometallic complex is crucial for the activity and that both ruthenium and iron are suitable as metallic atoms for this organometallic attachment. In series I, the ruthenocenyl group promoted a slightly stronger activity on adult schistosomes but also a slightly lower response on juvenile *S. mansoni* compared to the structurally identical derivative containing an iron instead of the ruthenium atom. Therefore, as both atoms seem to work equally as the central atom of the organometallic complex, their redox properties (that differ from Fc to Rc) seem to be of little importance. On the contrary, the distance between the organometallic complex and the niridazole structure seems to be of more importance. Structurally, the only difference between the compounds **A3** and **A4** is the length of the alkyl chain attaching the organometallic complex to the 2‐imidazolidone ring. In the active compound **A3**, the ferrocenyl complex is linked via one carbon atom, while the linker for compound **A** **4** is built of a six‐carbon atom chain, which leads to activity loss.

The potency against adult schistosomes improved through the attachment of the methylene ferrocenyl‐ or ruthenocenyl‐group by 1.6 and 1.8 times, respectively, compared to the parent compound niridazole.

The further modified ferrocenyl‐derivatives compounds **A2** and **A5** are both active against juvenile and adult schistosomes. For compound **A5**, potency was further increased by attaching the alkene group to the linking carbon atom between the organometallic complex and the 2‐imidazolidone ring. This ethenyl derivative gave the strongest drug response on adult schistosomes with an EC_50_ value of 1.5 µM, which reflects a 3.8‐fold stronger drug response than niridazole.

The attachment of the organometallic complex via a carbonyl group in compound **A** **2** broadened the activity spectrum by gaining pan activity against all life stages of *S. mansoni*. With an EC_50_ value of 2.0 µM after 72 h of incubation, compound **A2** was also the most active compound on *S. mansoni* NTS. The only other compounds with drug effects on NTS were the 2‐amino‐5‐nitrothiazole derivatives compounds C1 and D1 from series II. Compound **A2**, with its good activity against all developmental stages of schistosomes relevant in the human infection, together with its low cytotoxic potential and its species‐specific drug effect, is a very promising candidate. Since compound **A2** is also stable under warm and moist conditions (see Figure S36, Supporting Information), cheap in production due to a simple synthesis procedure, and suitable for oral administration, it fulfills all the required characteristics for an ideal antischistosomal that can be given as a preventive chemotherapeutic in MDA campaigns. Activity against other human pathogenic *Schistosoma* species like *S. haematobium*, *S. japonicum*, or *S. mekongi* remains to be investigated.

All compounds from series I, including niridazole itself, that were active against adults, were also active against juvenile schistosomes but lacked activity on NTS. This observation could be a result of the fact that NTS are not as metabolically active as later developmental stages,^[^ ^45^ ^,^ ^46^ ^]^ while niridazole is thought to be a prodrug and needs to be converted into its active metabolite(s) to unfold its drug effect on schistosomes.^[^ ^22^ ^,^ ^47^ ^]^

This hypothesis is further supported by the observation that all derivatives from series I, including niridazole itself, increase their effect on the schistosomes over time. This delayed onset of action could be due to the time required to build up the effective concentration of the active metabolite(s) through the metabolization of niridazole by the adult *S. mansoni*.^[^ ^22^ ^,^ ^48^, ^49^ ^–^ ^50^ ^]^

The series of I compounds were inactive in the in vitro evaluation of the cytotoxicity potential. None of the derivatives showed toxicity in any of the tested cell lines (human hepatocyte‐derived HUH7 and neuroblast‐derived SH‐SY5Y cells). It is important to highlight that niridazole itself showed no signs of toxicity in these cytotoxicity tests, despite the fact that it is already known that niridazole causes several safety issues.^[^ ^21^ ^,^ ^25^ ^,^ ^26^ ^,^ ^51^ ^]^ However, the applied cell models exhibit very low metabolic capacity toward xenobiotics. Primary hepatocytes and liver microsomal preparations may be applied to address the possibility that reactive metabolites cause toxicity. Further cytotoxic evaluations on niridazole and its derivatives could focus not only on the compounds themselves but also on their resulting metabolites, since previous studies already identified several metabolites of niridazole that are thought to cause different toxicities.^[^ ^47^ ^,^ ^52^, ^53^, ^54^, ^55^, ^56^, ^57^, ^58^ ^–^ ^59^ ^]^

In series II of the niridazole derivatives, the 2‐imidazolidone ring is replaced by different organometallic moieties attached to the nitrothiazole part of the niridazole molecule. The in vitro evaluation of the activity profile showed that for most derivatives, the activity against *S. mansoni* is lower with the loss of the second ring structure (2‐imidazolidone ring). Only compounds **C1** and **D1** showed activity from this series, revealing a different activity pattern than observed for the active series I compounds. While all series I compounds required 48 h to unfold their drug effect on adult schistosomes, compound **D1** was fast acting and showed an EC_50_ value below 10 µM already at the 24 h evaluation time point. This early onset of drug action is also unlike the activity profile we observed here for the parent compound niridazole.

The toxicity also deviates considerably for derivative series II. While no cytotoxicity was detected in series I, cytotoxic effects were found for three of the five compounds from series II. Compounds **A1**, **C1**, and **D1** showed a reduced XTT signal after 24 h of incubation at 20 µM in human SH‐SY5Y and HUH7 cells. Even though the signal reduction was more pronounced in the neuroblastoma cells, the reduced XTT signal was detected in both cell lines, indicating general toxicity. Since the signal reduction in the XTT assay can be due to direct cytotoxicity or indirectly by impairment of mitochondrial function, a SYTOX cell viability test was performed. Compounds **C1** and **D1** reduced cell viability, indicating cytotoxicity. Compound **A1** was inactive in the SYTOX assay. Whether mitochondria are impaired directly by compounds **A1**, **C1**, and **D1** or whether indirect toxicity leads to reduced mitochondrial capacity needs to be investigated.

## 4 Conclusion

The results presented in this study are a valuable supplement to the existing research on niridazole, which is very limited and outdated. Niridazole is a perfect example to demonstrate how drug development against schistosomiasis was heavily neglected once praziquantel was discovered and established as an improved and successful schistosomiasis treatment.^[^ ^60^ ^]^

The modification of the activity profile through introducing organometallic moieties was successful and led to different derivatives with improved or expanded activity profiles against *S. mansoni*. Future studies could involve in vivo characterization of compound **A** **2** which showed promising activity against all life cycle stages of *S. mansoni*. Other candidates for further studies would be the compounds **A3**, **A5**, and **B2** that showed higher potency against adult schistosomes. Since variances in sensitivity toward niridazole amongst different *Schistosoma* species are known, in vitro and later also in vivo tests on multiple *Schistosoma* species should be considered, to evaluate the activity profiles of the derivatives on different schistosome species. The information gained on the SAR of the different organometallic modifications is also valuable and could be used to design a second generation of organometallic derivatives of niridazole with further improved activity profiles.

## 5 Experimental Section

The experimental section for the chemistry and biology is presented in the Supporting Information.

## Conflict of Interest

The authors declare no conflict of interest.

## Author Contributions

**Tanja Karpstein**, **Sarah Keller**, **Shuai Zhong**, **Philipp Späne**, **Alex Odermatt**, **Jennifer Keiser** and **Gilles Gasser** conceived and designed the study. Synthesis and characterization of the compounds were performed by **Sarah Keller** and **Shuai Zhong** with assistance from **Kevin Cariou.** Biological experiments were carried out by **Tanja Karpstein** and **Cécile Häberli**, and resulting data analysis and presentation were done by **Tanja Karpstein** and **Gordana Panic**. Cytotoxic evaluations were performed by **Philipp Späne** and **Alex Odermatt**. X‐ray crystallography was carried out by **Olivier Blacque** All authors were involved with the interpretation of experiments and with the writing and the review editing of the manuscript. **Tanja Karpstein** drafted the first version of the manuscript with help from **Gordana Panic**. All authors have given approval to the final version of the manuscript. **Tanja Karpstein** and **Shuai Zhong** contributed equally to this work.

## Supporting information

Supplementary Material

## Data Availability

The data that support the findings of this study are available in the supplementary material of this article.
