# Supplementary material for: Multidisciplinary Preclinical Investigations on Ferrocenyl, Ruthenocenyl, and Benzyl Derivatives of Niridazole as New Drug Candidates against Schistosomiasis
Source: ChemMedChem. 2025 Nov 4;20(23):e202500603. doi: 10.1002/cmdc.202500603 (PMC12677843; doi:10.1002/cmdc.202500603)
Supplement: Supplementary file 1 — Supplementary Material [file CMDC-20-e202500603-s001.pdf]

Multidisciplinary preclinical investigations on ferrocenyl, ruthenocenyl and benzyl derivatives of niridazole as new drug candidates against schistosomiasis

Tanja Karpstein,[1,2,#] Shuai Zhong,[3,#] Sarah Keller,[3] Philipp Späne,[4] Cécile Häberli,[1,2] Gordana Panic,[1,2] Olivier Blacque,[5] Alex Odermatt,[4,6] Kevin Cariou,[3] Gilles Gasser,[3,] and Jennifer Keiser[1,2,*]*

[1] Department of Medical Parasitology and Infection Biology, Swiss Tropical and Public Health Institute, Kreuzstrasse 2, CH-4123 Allschwil, Switzerland

[2] University of Basel, CH-4003, Basel, Switzerland

[3] Chimie ParisTech, PSL University, CNRS, Institute of Chemistry for Life and Health Sciences, Laboratory for Inorganic Chemical Biology, 75005 Paris, France.

[4] Division of Molecular and Systems Toxicology, Department of Pharmaceutical Sciences, University of Basel, Klingelbergstrasse 50, 4056 Basel, Switzerland.

[5] Department of Chemistry, University of Zurich, 8001 Zurich, Switzerland.

[6] Swiss Centre for Applied Human Toxicology and Department of Pharmaceutical Sciences, University of Basel, Missionsstrasse 64, 4055 Basel, Switzerland.

[#] have contributed equally to the work

[*] Corresponding Authors: E-mail:

kevin.cariou@chimieparistech.psl.eu;gilles.gasser@chimieparistech.psl.eu;

jennifer.keiser@swisstph.ch.

Content

1. Experimental section for synthesis........................ S Error! Bookmark not defined.

1.1 Materials and methods.................................... S Error! Bookmark not defined.

1.2 Synthetic procedures and compound characterization S Error! Bookmark not defined.

2. Experimental section for biological experiments . S Error! Bookmark not defined.

2.1 *In vitro* evaluations in helminths...................... SError! Bookmark not defined.

2.3 Cytotoxic evaluations....................................... S Error! Bookmark not defined.

3. NMR spectra of compounds.................................. S Error! Bookmark not defined.

4. Stability assays.. S32

5. X-ray Crystallography S35

6. References for the supplementary S42

1. Experimental section for synthesis

1.1 Materials and methods

5-Nitrothiazol-2-amine, 2-Chloroethyl isocyanate, Bis(cyclopentadienyl)ruthenium(II) and 1-[Bis(dimethylamino)methylene]-1H-1,2,3-triazolo[4,5-b]pyridinium 3-oxide hexafluorophosphate (HATU) were obtained from BLD Pharmatech. Celite 281, 1-Adamantanecarboxylic acid, n-Butyllithium (2.5 M in hexanes), Iodomethane, tert-Butyllithium (1.7 M in pentane) and *N,N*-Dimethylmethyleneiminium chloride were purchased from Sigma-Aldrich. Potassium carbonate, Ammonium chloride, *N,N*-Dimethylmethyleneiminium iodide, *N,N*-dimethylformamide (DMF, 99.8%, Extra Dry, AcroSeal™) and Sodium bicarbonate were purchased from Thermo-Scientific. Methyltriphenylphosphonium iodide, Benzoic acid, *N,N*-diisopropylethylamine (DIPEA) and Ferrocene were purchased from TCI Chemicals. Sodium sulfate and potassium tert-butoxide were purchased from Alfa Aesar. Ferrocenecarboxylic acid and (Dimethylaminomethyl)ferrocene were purchased from Ambeed Inc. Chloroform-*d*, Dimethyl sulfoxide (DMSO)-d_6, Acetone-d_6 and Acetonitrile-d_3 were ordered in Eurisotop. All solvents were purchased of analytical or HPLC grade from CARLO ERBA Reagents, and solvents for reactions were either of pro analysis (p.a.) grade or distilled prior to their use. Evaporation of solvents in vacuo was done with a rotary evaporator at 40 ° C. Thin layer chromatography (TLC) was performed using silica gel 60 F-254 (Merck) plates with detection of spots being achieved by exposure to UV light. NMR spectra were recorded in deuterated solvents on Bruker Avance-400 and Avance-Neo 500 spectrometers at room temperature. The chemical shifts, δ, are reported in ppm (parts per million). The abbreviations for the peak multiplicities are as follows: s (singlet), d (doublet), t (triplet), dd (doublet of doublets), brs (broad-), and m (multiplet). High-resolution ESI mass spectrometry (HR ESI-MS) spectra were recorded on an LTQ-Orbitrap XL from Thermo Scientific.

1.2 Synthetic procedures and compound characterization

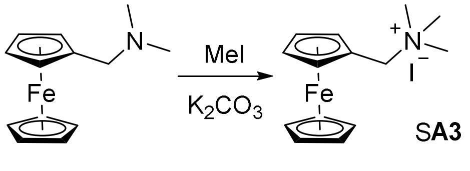

S**A3**

Compound S**A3** was prepared following an adapted literature procedure. Yield: 95%. **^{1}H NMR** (400 MHz, D_2O) δ 4.56 (s, 2H), 4.48 (s, 2H), 4.43 (s, 2H), 4.32 (s, 5H), 2.99 (s, 9H). The spectral data corresponds to the previously reported data.[1]

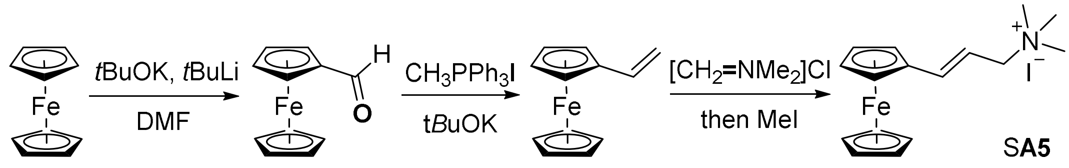

Compound S**A5** was prepared following an adapted literature procedure. Yield: 34%. **^{1}H NMR** (400 MHz, Chloroform-*d*) δ 6.95 (d, *J* = 15.4 Hz, 1H), 5.83 (dt, *J* = 15.4, 7.8 Hz, 1H), 4.45 – 4.44 (m, 2H), 4.40 (d, *J* = 7.8 Hz, 2H), 4.34 – 4.33 (m, 2H), 4.14 (s, 5H), 3.41 (s, 9H) ppm. The spectral data corresponds to the previously reported data.[2] For the Ferrocene carboxaldehyde, **^{1}H NMR** (400 MHz, Chloroform-*d*) δ 9.96 (s, 1H), 4.80 (s, 2H), 4.61 (s, 2H), 4.28 (s, 5H) ppm and Vinylferrocene, **^{1}H NMR** (400 MHz, Chloroform-*d*) δ 6.45 (dd, *J* = 17.5, 10.7 Hz, 1H), 5.34 (dd, *J* = 17.4, 1.2 Hz, 1H), 5.04 (dd, *J* = 10.7, 1.2 Hz, 1H), 4.36 – 4.35 (m, 2H), 4.21 – 4.20 (m, 2H), 4.11 (s, 5H) ppm.

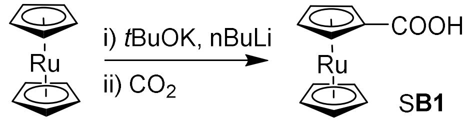

Compound S**B1** was prepared following an adapted literature procedure. Yield: 76%, 415 mg. **^{1}H NMR** (400 MHz, Chloroform-*d*) δ 5.17 – 5.16 (m, 2H), 4.75 – 4.73 (m, 2H), 4.63 (s, 5H). The spectral data corresponds to the previously reported data.[3]

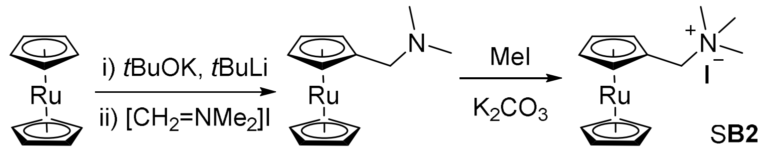

Compound S**B2** was prepared following an adapted literature procedure.[1,4] Yield: 51%. **^{1}H NMR** (400 MHz, Chloroform-*d*) δ 4.89 – 4.88 (m, 2H), 4.70 – 4.69 (m, 2H), 4.65 (s, 5H), 4.55 (s, 5H), 3.36 (s, 9H). For the dimethylamino-methylruthenocene, ^{1}H NMR (400 MHz, Chloroform-*d*) δ 4.55 (s, 2H), 4.46 (s, 5H), 3.05 (s, 2H), 2.16 (s, 6H) and the spectral data correspond to the previously reported data.[3]

General Procedure A[5]

Anhydrous DMF (3.0 mL) was added under N_2 atmosphere to a flask containing the appropriate carboxylic acid (1.2 eq.) and 2-Amino-5-nitrothiazole (29 mg, 0.2 mmol), followed by the addition of and *N*, *N*-diisopropylethylamine (0.6 mmol). After being stirred for 15 min at room temperature, HATU (1.2 eq.) was added to the reaction mixture and stirred overnight at room temperature under N_2 atmosphere. Upon completion of the reaction, the DMF was removed in vacuum. Saturated aqueous

NH_4Cl (10 ml) was added, and the mixture was extracted with CH_2Cl_2 (3 x 10 ml). The CH_2Cl_2 layer was washed with saturated aqueous $NaHCO_3$ and dried with anhydrous Na_2SO_4. The solvent was removed in vacuum and resulting crude material was purified by column chromatography.

General Procedure B[6]

In a 25 mL round-bottle flask, the ferrocenecarboxylic acid (1.1 eq.) and TBTU (1.1 eq.) dissolved in THF (10 mL), followed by the addition of DIPEA (3.0 eq.). After being stirred for 30 min at room temperature, Niridazole (1.0 eq.) was added to the solution and reaction mixture was stirred overnight at room temperature. After the reaction, the solvent was removed in vacuo then washed with brine (20 mL), the product was extracted with CH_2Cl_2 (3 x 20 mL). The layers were separated, and the organic layer was dried over anhydrous Na_2SO_4, filtered and concentrated in vacuum to afford the crude product.

General Procedure C[7]

Niridazole (1.0 eq) and potassium carbonate (3.0 eq) were added to a round bottom flask then suspended in dry acetonitrile (50 mL, de-gas with N_2), followed by the addition of ligand (1.5 eq). The reaction mixture was refluxed overnight in dry acetonitrile under N_2 atmosphere. Upon cooling to RT, the mixture was filtered through a paper filter and all volatiles were removed in vacuo. The product was purified via column chromatography.

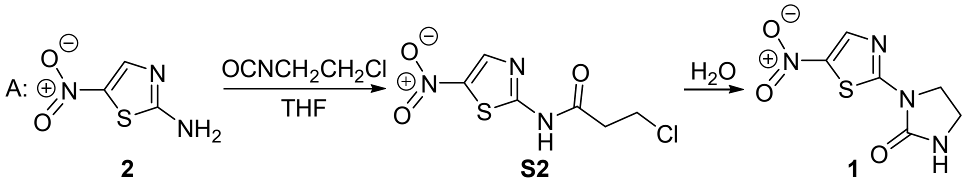

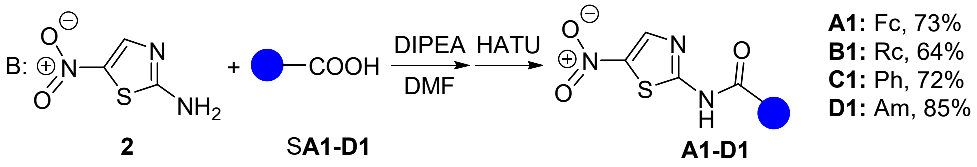

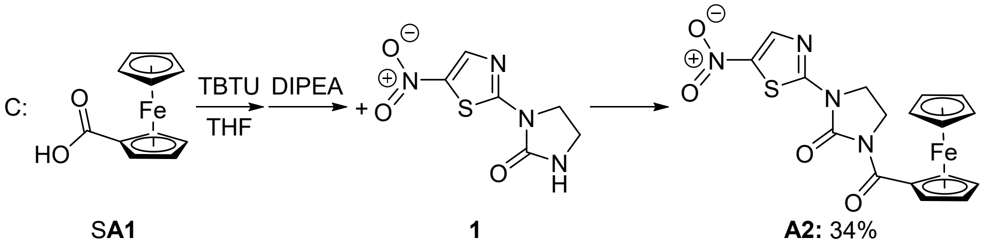

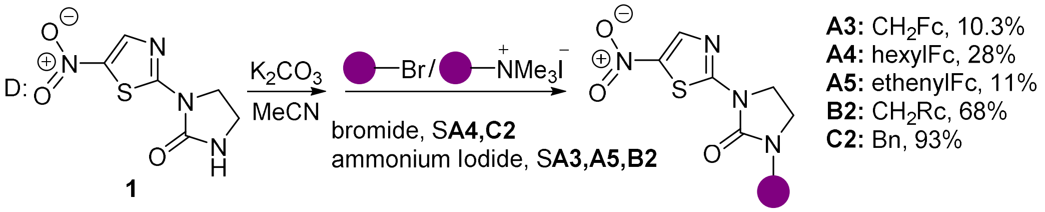

Scheme **S1**. Synthesis of the Analogs of Niridazole. Reagents and conditions: A) THF, reflux, 48 h then H_2O, 85 °C, 12 h; B) DIPEA, HATU, DMF, RT, overnight; C) DIPEA, TBTU, THF, RT, overnight; D) K_2CO_3, MeCN, reflux, overnight.

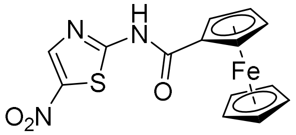

Compound **A1** was prepared according to the general procedure **A** using 2-Amino-5-nitrothiazole **2** (0.2 mmol) and ferrocenecarboxylic acid S**A1** (0.24 mmol), the crude product was purified by column chromatography (SiO_2) with cyclohexane and ethyl acetate (2:1). Yield: 73%. **^{1}H NMR** (400 MHz, Acetonitrile-d_3) δ 10.29 (brs, 1H, NH), 8.41 (s, 1H), 5.02 (t, J = 2.0 Hz, 2H), 4.62 (t, J = 2.0 Hz, 2H), 4.27 (s, 5H) ppm. **^{13}C NMR** (100 MHz, Acetonitrile-d_3) δ 162.5, 142.5, 73.1 (*Fc*-CH_2), 70.8 (Fc), 69.8 (*Fc*-CH_2) ppm. **Molecular wt**: 357.1650 g·mol^{-1}. **HRMS** (ESI) m/z: calculated [M]$^-$ 355.9792; found [M]$^-$ 355.9786 (NH deprotonated for negative ionization). **Elemental Analysis**: $C_{14}H_{11}FeN_3O_3S$ Calculated C 47.08, H 3.10, N 11.77; Found C 47.65, H 2.82, N 11.73.

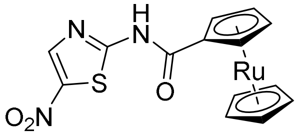

Compound **B1** was prepared according to the general procedure **A** using 2-Amino-5-nitrothiazole **2** (0.2 mmol) and benzoic acid S**B1** (0.24 mmol), the crude product was purified by column chromatography (SiO_2) with cyclohexane and ethyl acetate (2:1). Yield: 64%. **^{1}H NMR** (400 MHz, Chloroform-*d*) δ 9.13 (brs, 1H, NH), 8.30 (s, 1H), 5.18 (t, J = 1.8 Hz, 2H), 4.84 (t, J = 1.8 Hz, 2H), 4.63 (s, 5H) ppm. **^{13}C NMR** (100 MHz, Chloroform-*d*) δ 140.8, 74.0 (*Rc*-CH_2), 72.6 (Rc), 70.2 (*Rc*-CH_2) ppm. **Molecular wt:** 402.3900 g·mol^{-1}. **HRMS** (ESI) m/z: calculated [M]$^-$ 401.9486; found [M]$^-$ 401.9490 (NH deprotonated for negative ionization). **Elemental Analysis**: $C_{14}H_{11}N_3O_3RuS$ Calculated C 41.79, H 2.76, N 10.44; Found C 41.53, H 2.63, N 10.31.

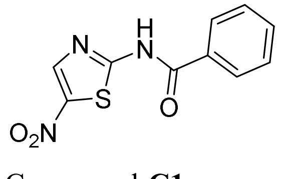

Compound **C1** was prepared according to the general procedure **A** using 2-Amino-5-nitrothiazole **2** (0.2 mmol) and benzoic acid S**C1** (0.24 mmol), the crude product was purified by column chromatography (SiO_2) with cyclohexane and ethyl acetate (1:1). Yield: 72%. Also commercially acquired from TCI and used without further purification. **^{1}H NMR** (400 MHz, Acetone-d_6) δ 8.40 (s, 1H), 8.12 (dd, J = 8.5, 1.2 Hz, 2H), 7.65 – 7.61 (m, 1H), 7.54 – 7.50 (m, 2H). **^{13}C NMR** (100 MHz, Acetone-d_6) δ 209.9, 166.9, 163.0, 142.3, 134.3, 131.9, 129.7, 129.1. **Elemental Analysis**: $C_{10}H_7N_3O_3S$ Calculated C 48.19, H 2.83, N 16.86; Found C 48.18, H 2.50, N 16.67.

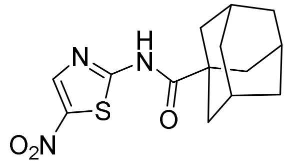

Compound **D1** was prepared according to the general procedure **A** using 2-Amino-5-nitrothiazole **2** (0.2 mmol) and 1-Adamantanecarboxylic acid S**D1** (0.24 mmol), the crude product was purified by column chromatography (SiO_2) with cyclohexane and ethyl acetate (2:1). Yield: 85%. **^{1}H NMR** (400 MHz, Acetone-d_6): δ 8.38 (s, 1H), 2.06 – 2.04 (m, 9H), 1.76 (m, 6H) ppm. **^{13}C NMR** (100 MHz, Acetone-d_6): δ 177.9 (Cq, C=O), 162.9, 143.5, 142.3, 42.1 (Cq, adamantyl), 38.6 (adamantyl), 36.7 (adamantyl), 28.7 (adamantyl) ppm. **Molecular wt**: 307.3680 g·mol^{-1}. **HRMS** (ESI) m/z: calculated [2M-H]$^-$ 613.1908; found [2M-H]$^-$ 613.1910 (one NH of two molecules deprotonated for negative ionization). **Elemental Analysis**: $C_{14}H_{17}N_3O_3S$ Calculated C 54.71, H 5.58, N 13.67; Found C 54.53, H 5.64, N 13.15.

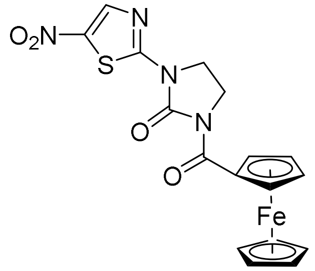

Compound **A2** was prepared according to the general procedure **B** using Niridazole **1** (0.2 mmol) and ferrocenecarboxylic acid S**A1** (0.24 mmol), the crude product was purified by column chromatography with heptane and ethyl acetate (9:1). Yield: 34%. **^{1}H NMR** (400 MHz, Acetone-d_6): δ 8.45 (s, 1H), 5.06 (t, J = 2.0 Hz, 2H), 4.55 (t, J =

2.0 Hz, 2H), 4.32 – 4.30 (m, 2H), 4.28 – 4.26 (m, 2H), 4.24 (s, 5H, Fc) ppm. **^{13}C NMR** (125 MHz, Acetone-d_6): δ 142.4, 74.6, 72.9 (*Fc*-CH$_2$), 71.9 (*Fc*-CH$_2$), 71.0 (Fc), 42.9, 42.3 ppm. **Molecular wt**: 426.2280 g·mol^{-1}. **HRMS** (ESI) m/z: calculated [M]$^+$ 426.0085; found [M]$^+$ 426.0079. **Elemental Analysis**: $C_{17}H_{14}FeN_4O_4S$ Calculated C 47.91, H 3.31, N 13.15; Found C 48.09, H 3.12, N 13.12.

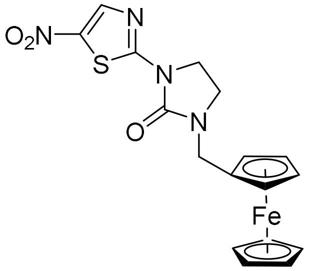

Compound **A3** was prepared according to the general procedure **C** using Niridazole (100 mg, 0.47 mmol) and potassium carbonate (190 mg, 1.37 mmol, 2.9 eq) in a round bottom Schlenk flask. The compounds were suspended in dry acetonitrile (50 mL) and (Ferrocenylmethyl)trimethylammonium Iodide (270 mg, 0.70 mmol, 1.5 eq) was added. The crude product was purified by column chromatography with hexane and ethyl acetate. Yield: 10.3%. ^{1}H NMR (400 MHz, Acetone-d_6): δ 8.34 (s, 1H, aromat.), 4.33 (s, 2H, C*H$_2$*-Fc), 4.30 (t, J = 1.8 Hz, 2H, *Fc*-CH$_2$), 4.21 (s, 5H, Fc), 4.17 (t, J = 1.8 Hz, 2H, *Fc*-CH$_2$), 4.15 – 4.11 (m, 2H), 3.69 – 3.65 (m, 2H) ppm. **^{13}C NMR** (100 MHz, Acetone-d_6): δ 163.1, 155.2, 143.1, 82.9, 70.0, 69.5, 69.3, 69.0, 68.8, 44.1, 42.9, 42.8 ppm. **Molecular wt**: 412.2450 g·mol^{-1}. **HRMS** (ESI) m/z: calculated [M]$^+$ 412.0293; found [M]$^+$ 412.0286 (Fe(II) oxidized to Fe(III) for positive ionization). **Elemental Analysis**:$C_{17}H_{16}FeN_4O_3S \cdot 0.5\ H_2O$ Calculated C 48.47, H 4.07, N 13.30; Found C 48.36, H 3.75, N 13.93.

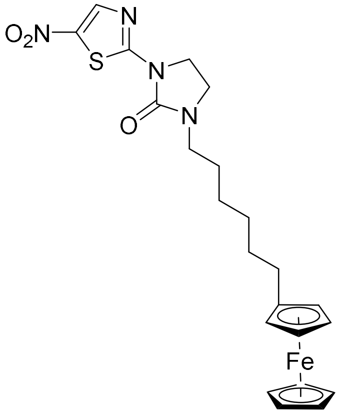

Compound **A4** was prepared according to the general procedure **C** using Niridazole (100 mg, 0.47 mmol) and potassium carbonate (193 mg, 1.40 mmol) were added to a

round bottom Schlenk flask. The compounds were suspended in dry acetonitrile (50 mL) and (6-bromhexyl)-ferrocene (244 mg, 0.70 mmol) was added. The crude product was purified by column chromatography with hexane to ethyl acetate (3:1). Yield: 28%. **^{1}H NMR** (400 MHz, Acetone-d_6): δ 8.35 (s, 1H), 4.21 – 4.17 (m, 2H), 4.08 (s, 5H), 4.07 (s, 2H), 4.01 (s, 2H), 3.80 – 3.76 (m, 2H), 3.36 (t, J = 7.2 Hz, 2H, Fc-(CH_2)$_6$), 2.35 (t, J = 7.6 Hz, 2H), 1.65 – 1.60 (m, 2H, Fc-(CH_2)$_6$), 1.55 – 1.51 (m, 2H, Fc-(CH_2)$_6$), 1.41 – 1.37 (m, 4H, Fc-(CH_2)$_6$) ppm. **^{13}C NMR** (100 MHz, Acetone-d_6): δ 143.1, 69.2 (Fc), 68.8 (*Fc*-CH$_2$), 67.7 (*Fc*-CH$_2$), 44.3 (Fc-(CH_2)$_6$), 43.0, 42.8, 31.8 (Fc-(CH_2)$_6$), 27.8 (Fc-(CH_2)$_6$), 27.2 (Fc-(CH_2)$_6$) ppm. **Molecular wt**: 482.3800 g·mol^{-1}. **HRMS** (ESI) m/z: calculated [M]$^+$ 482.1075; calculated [M]$^+$ 482.1070. **Elemental Analysis**: $C_{22}H_{26}FeN_4O_3S$ Calculated C 54.78, H 5.43, N 11.61; Found C 55.04, H 5.33, N 11.36.

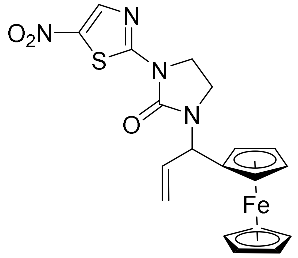

Compound **A5** was prepared according to the general procedure **C** using Niridazole (60 mg, 0.28 mmol) and potassium carbonate (116 mg, 0.84 mmol) were added to a round bottom Schlenk flask. The compounds were suspended in dry acetonitrile (50 mL) and (Ferrocenylpropyl-1-en) trimethylammonium Iodide (173 mg, 0.42 mmol) was added. The unexpected product was purified by column chromatography with heptane and ethyl acetate (9:1). Yield: 11%. **^{1}H NMR** (400 MHz, Acetone-d_6): δ 8.35 (s, 1H), 6.41 – 6.33 (m, 1H), 5.54 (d, J = 5.8 Hz, 1H), 5.44 – 5.39 (m, 1H), 5.37 – 5.34 (m, 1H), 4.24 (s, 5H), 4.16 – 4.12 (m, 2H), 3.67 – 3.61 (m, 1H), 3.38 – 3.31 (m, 1H), 2.78 (t, J = 1.0 Hz, 4H) ppm. **^{13}C NMR** (100 MHz, Acetone-d_6): δ 143.00, 117.66, 69.80, 69.45, 68.81, 68.22, 55.43, 42.85, 39.48 ppm. **Molecular wt**: 438.2830 g·mol^{-1}. HRMS (ESI) m/z: calculated [M]$^+$ 438.0444; found [M]$^+$ 438.0445. $C_{19}H_{18}FeN_4O_3S{\cdot}0.5(CD_3)_2O{\cdot}2.0H_2O$ Calculated C 48.63, H 4.38, N 11.07; Found C 47.08, H 3.10, N 11.77.

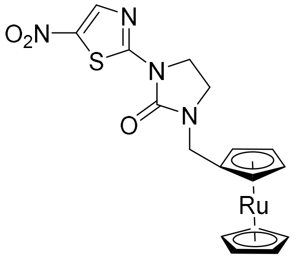

Compound **B2** was prepared according to the general procedure **C** using Niridazole (60 mg, 0.28 mmol) and potassium carbonate (116 mg, 0.84 mmol) in a round bottom Schlenk flask. The compounds were suspended in dry acetonitrile (50 mL) and (Ruthenocenylmethyl)trimethylammonium Iodide (181 mg, 0.42 mmol) was added. The crude product was purified by column chromatography with cyclohexane and ethyl acetate (3:2). Yield: 68%. **^{1}H NMR** (400 MHz, Acetone-d_6): δ 8.35 (s, 1H, aromat.), 4.71 (t, J = 1.7 Hz, 2H, *Rc*-CH_2), 4.58 (s, 5H, Rc), 4.53 (t, J = 1.7 Hz, 2H, *Rc*-CH_2), 4.19 – 4.14 (m, 2H), 4.14 (s, 2H, CH_2-Rc), 3.81 – 3.76 (m, 2H) ppm. **^{13}C NMR** (100 MHz, Acetone-d_6): δ 163.1, 155.1, 143.0, 82.8, 72.0, 71.6, 71.1, 43.8, 43.0, 42.8 ppm. **Molecular wt**: 457.4700 g·mol^{-1}. **HRMS** (ESI) m/z: calculated $[M+H]^+$ 459.0065; found $[M+1H]^+$ 459.0059 (Ru does not get oxidized). **Elemental Analysis**: $C_{17}H_{16}N_4O_3RuS$ Calculated C 44.63, H 3.53, N 12.25; Found C 44.95, H 3.24, N 11.80.

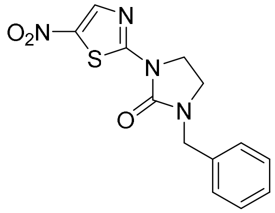

Compound **C2** was prepared according to the general procedure **C** using Niridazole (60 mg, 0.28 mmol) and potassium carbonate (116 mg, 0.84 mmol) in a round bottom Schlenk flask. The compounds were suspended in dry acetonitrile (50 mL) then benzyl bromide (0.05 mL, 0.42 mmol) were added. The crude product was purified by column chromatography with cyclohexane and ethyl acetate then washing with pentane. Yield: 93%. **^{1}H NMR** (400 MHz, Acetone-d_6): δ 8.36 (s, 1H, aromat.), 7.39 – 7.32 (m, 5H), 4.56 (s, 2H), 4.22 – 4.18 (m, 2H), 3.70 – 3.66 (m, 2H) ppm. **^{13}C NMR** (100 MHz, Acetone-d_6): δ 163.1, 155.1, 143.0, 72.0, 71.6, 71.1, 43.8, 43.0, 42.8 ppm. **Molecular wt**: 304.3240 g·mol^{-1}. **HRMS** (ESI) m/z: calculated $[M+Na]^+$ 327.0528; found $[M+Na]^+$ 327.1414; calculated $[M+H]^+$ 305.0708; found $[M+H]^+$ 305.0704. **Elemental Analysis**: $C_{13}H_{12}N_4O_3S\cdot 0.5\ H_2O$ Calculated C 49.83, H 4.18, N 17.88; Found C 50.33, H 3.79, N 17.39.

2. Experimental section for biological experiments

Stock solutions of the test compounds

Stock solution of a concentration of 10 mM in pure DMSO (Sigma-Aldrich, Switzerland) were prepared of the 12 compounds, these stock solutions were used for all *in vitro* evaluations from activity tests in *S. mansoni* to the cytotoxic determinations in HUH7 hepatocyte-derived carcinoma cells, and in SH-SY5Y neuroblastoma cells. The solutions were thoroughly vortexed and sonicated to enable a good dissolution of the compounds and homogenous stock solutions.

2.1 *In vitro* evaluations in *S. mansoni*

In vitro evaluation on newly transformed schistosomula (NTS)

To obtain newly transformed schistosomula (NTS) the lifecycle of *S. mansoni* (strain originates from West Africa, Liberia) is maintained at the Swiss Tropical and Public Health Institute (Swiss TPH). As previously described, cercariae are collected from infected *Biomphalaria glabrata* snails and then mechanically transformed into NTS.[8] After HBSS (Gibco, Waltham MA, USA) washing to get rid of the detached tails, the NTS were kept overnight (min. 12 h, max. 24 h) in culture medium at 37 °C and 5 % CO_2 to complete their transformation process, before the drug sensitivity assay was started. The drug assays were performed in the same culture medium consisting of medium M199 (Gibco, Waltham MA, USA) supplemented with 5 % v/v inactivated fetal calf serum (iFCS; Bioconcept AG, Allschwil, Switzerland), 1% antibiotics mixture developed by Mäser et al.,[9] and 1 % penicillin 10,000 U/mL, and streptomycin 10 mg/mL solution (Sigma-Aldrich, Buchs, Switzerland). The assay was performed in a 96-well plate, with 30−40 NTS per well in a final volume of 250 µL. NTS incubated in culture medium containing 0.5 % DMSO but no drug served as negative controls. Microscopic evaluation was performed after 24, 48 and 72 h of incubation of the parasites at 37 °C and 5 % CO_2. Their overall viability was scored from 0 (death) to 3 (full viability) by a phenotypic screening method based on their motility, morphology, and granularity.[8] All 12 compounds were initially tested twice at 50 µM in triplicate on NTS, identified hits with a mean drug effect of ≥ 60 % at 50 µM were further evaluated in a 1:2 serial dilution from 50 to 3.125 µM for EC_{50} determination.

In vitro evaluation on juvenile *S. mansoni*

Juvenile worms were gained through cultivation of NTS as previously described.[10] In short, NTS were obtained as described above, then directly after the mechanical transformation process the newly transformed schistosomula were placed in a special culturing medium. The culture medium consists of Panserin 401, w: L-Glutamine, serum free all-round medium (Pan Biotech, Aidenbach, Germany) supplemented with 20 % human serum and 500 U/mL penicillin, 500 mg/mL streptomycin (LuBioScience, Switzerland). 96-well transparent polystyrene flat bottom plates were used for the *in vitro* growth procedure, with 40 to 60 NTS per well in 100 µL culture medium. The bordering wells of the plate were filled solely with medium to counteract evaporation and to keep the humidity as steady as possible. The plates were incubated for 21 days at 5 % CO_2 and at 37 °C, with regular medium changes every 3 to 4 days. After the 21 days culturing period, the schistosomulae developed into juveniles. For the drug sensitivity assay, juvenile worms were incubated over 72 h (5 % CO_2 and 37 °C) with the test compound in the same medium, as used for the culturing process. In a 96-well plate, 250 µL medium containing the test compound and six juvenile worms, ideally of both sexes were placed in each well. All compounds were tested twice independently at 50, 10, 5 and 1 µM in duplicates. The viability of the worms was evaluated microscopically after 24, 48 and 72 h of continuous drug exposure using the same phenotypic screening method as described before.

In vitro* evaluation on adult *S. mansoni

After a week for acclimatization to the new environment with controlled conditions (temperature: 22 °C ± 2 °C, humidity: 50 % ± 10 %, 12:12 h light/dark photocycle), four-week-old female NMRI mice (Charles River, Sulzfeld, Germany) were subcutaneously infected with approximately 100 cercariae to establish the *S. mansoni* infection. In the *S. mansoni* infected mice the worms were allowed to develop for 7 weeks into the adult stage, before they were extracted from the mesenteric veins of the euthanized mice. The adult worms were placed into RPMI 1640 (Gibco, Waltham MA, USA) medium supplemented with 1 % penicillin/streptomycin mixture and 5 % iFCS at 37 °C and 5 % CO_2. The drug sensitivity assay was set up in a 24-well plate with two wells containing each two pairs of *S. mansoni* worms per compound and concentration. The final volume per well was 2 mL, containing the culture medium and the corresponding amount of drug dissolved in DMSO. The maximum concentration tested was 50 µM, corresponding to 0.5 % DMSO, to which all the other wells were adapted

to, so that all test concentrations contained always the same amount of DMSO. Additionally, 2 wells with negative controls containing only DMSO and the culture medium were included. After an incubation period of 72 h at 37 °C and 5 % CO_2, the worms were microscopically evaluated. The drug effect was determined 24, 48 and 72 h post-exposure based on the phenotype of the adult worms, using a viability scale ranging from three referring to viable, motile worms with no change in their tegument to zero standing for the death of the worms. Active compounds (mean drug effect of ≥ 60 %) at 50 μM were further evaluated at 10 μM, the ones that still showed mean drug effects ≥ 60 %, were also tested at 5 and 1 μM, to enable EC_{50} determination.

Ethics

All described *in vitro* studies with mice involved, were conducted at the Swiss Tropical and Public Health Institute, first in Basel, later in Allschwil, and had the approval by the corresponding veterinary authorities of the Canton Basel-Stadt (permit no. 2070) and the Canton Basel-Landschaft (permission no. 520 and 545) based on Swiss cantonal and national regulations.

2.3 Cytotoxic evaluations

Cell culture

HUH7 cells were obtained from ATCC (USA) and grown in DMEM - high glucose (Sigma Aldrich Cat D5796), supplemented with 10 % FBS (Biowest Cat. S140) and 100 U/mL penicillin/streptomycin (Sigma CAT P4333). SH-SY5Y cells were purchased from ATCC and grown in a mix of MEM (Sigma Aldrich cat:51416C) and F12 (Gibco Ref 21765) (1:1), supplemented with 10% FBS, 100 U/mL penicillin/streptomycin and 2 mM L-glutamine (Merck Cas 56-85-9 CAT 100286). Cells were regularly tested for the absence of mycoplasma.

XTT viability assay

To evaluate the cytotoxic potential of the compounds, the 2,3-bis(2-methoxy-4-nitro-5-sulfophenyl)-2H-tetrazolium-5-carboxanilide (XTT) assay was performed on SH-SY5Y neuroblastoma cells and HUH7 hepatocarcinoma cells. For both cell lines, 30’000 cells were seeded per well in a 96-well plate format in their individual complete growth medium. Cells were allowed to attach overnight. The media was exchanged to

serum-free media containing either treatment at a concentration of 20 µM or DMSO (ITW Reagents Cat A3672) control (0.2 %).
Following 24 h of incubation, 12.5 µL of an XTT/phenazine methosulfate (PMS) (XTT Sigma Aldrich cat X4626, PMS Serva CAT: 3203) 0.02solution (1 mg/mL XTT and 7.5 µg/mL PMS) was added to each well. Absorbance was measured using a microplate reader (Biotek Synergy HT) at 450 nm (signal) and 650 nm (background), 2 h after the addition of the XTT/PMS solution. Individual backgrounds were subtracted and all results were normalized to DMSO control. Mean ± SD was determined from three independent experiments, each performed in quintuplicate.

SYTOX cell viability assay

The cytotoxic potential was additionally evaluated using the staining-based SYTOX viability assay. SH-SY5Y cells were seeded at a density of 15'000 cells per well in a 96-well plate in complete growth medium. After overnight attachment, the medium was exchanged for treatment to serum-free and charcoal-treated medium (Sigma Aldrich cat C9157) containing 20 µM of compound or DMSO control (0.2 %). To obtain serum-free and charcoal-treated media, serum-free growth media was mixed with charcoal (50 mL per 1 g of charcoal) for 1 h at 4°C and centrifuged at 6000 g for 15 minutes. The supernatant was sterile filtered (0.20 µm). Following 24 h of incubation, cells were stained by adding SYTOX Green (Invitrogen cat S7020) and Höchst (Invitrogen cat 33342) to achieve a final concentration of 1 µM (SYTOX Green) and 5 µg/mL (Höchst) for 60 min. Data was acquired on the BioTeck Cytation5 microscope and evaluated by the manufacturer's software (Gen5 Image Prime Version 3.12). Nuclei count (Höchst positive) and cell viability (SYTOX Green negative) was established by evaluating 16 fields per well on a 20x objective utilizing the appropriate filters. Mean ± SD was determined from three independent experiments, each performed in quadruplicate.

Data Analysis

Drug effects from the *S. mansoni in vitro* assays were calculated by normalizing the mean viability values (±SEM) of treated larvae, NTS, juveniles or adult worms to the control parasite viability values. The EC_{50} values were calculated using GraphPad Prism (Version 8.2.1) from the *in vitro* screening data of at least four different test concentrations. IC_{50} values against the mammalian cell line (L6) were generated using the Softmax software (SoftMax pro v 5.4.6.005) and Excel 2016. Selectivity indices

(SIs) were calculated by dividing the L6 cytotoxicity specified as IC_{50} value, by the helminth activity given as EC_{50} value. GraphPad Prism was used to analyze the data of the XTT and SYTOX tests.

Table S1: *In vitro* activity at 10 µM of the seven active compounds on adult *S. mansoni.* Incubation for 72 h at 37 °C and 5 % CO_2 was performed on adult *S. mansoni* with either compound (10 µM) or DMSO (0.1 %). The schistosome's viability was assessed microscopically and scored from 0 to 3 every 24 h. Drug effects are shown as mean ± SD in percentage of the DMSO control from two independent experiments each performed in duplicate. The cut-off criteria for classification as active compound was set as drug effect of at least 60 %.

	24h		48h		72h	
**Compound**	**mean drug effect [%]**	**SD**	**mean drug effect [%]**	**SD**	**mean drug effect [%]**	**SD**
**B1**	45.53	56.95	44.35	32.69	57.20	33.78
**C1**	49.58	43.23	50.37	39.32	58.15	30.78
**D1**	55.18	41.04	66.75	18.73	65.76	20.52
**A2**	36.97	32.34	46.61	37.53	56.40	34.51
**A3**	30.75	42.93	44.28	25.73	66.09	29.99
**A5**	36.19	25.18	57.59	49.40	99.09	10.91
**B2**	31.28	23.80	44.99	24.88	65.57	46.42

3. NMR spectra of compounds

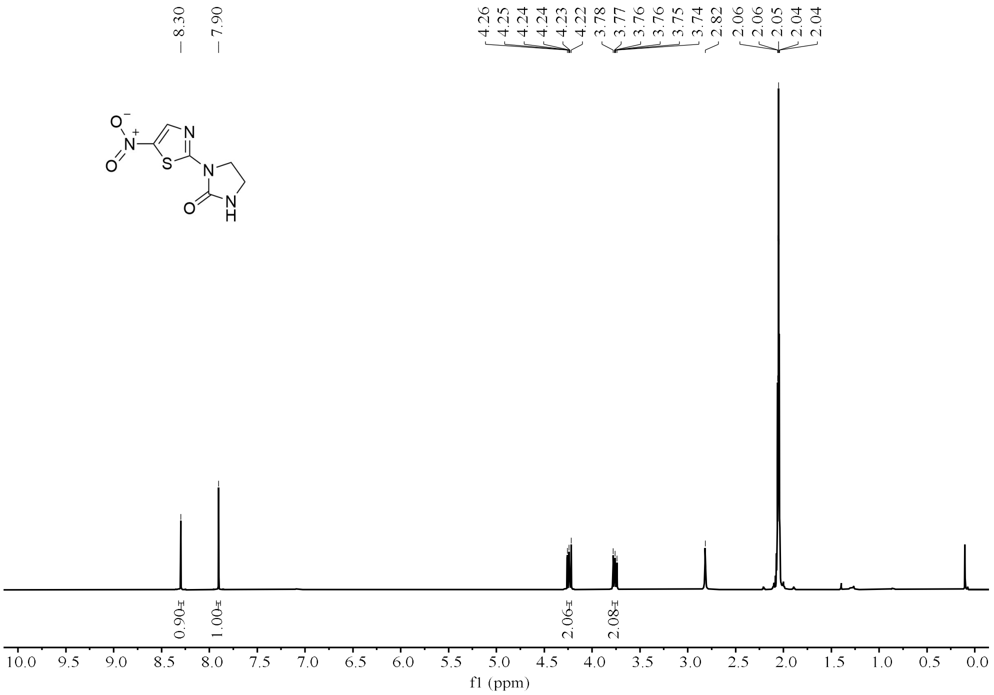

Figure S1 ^{1}H NMR spectrum of **1**

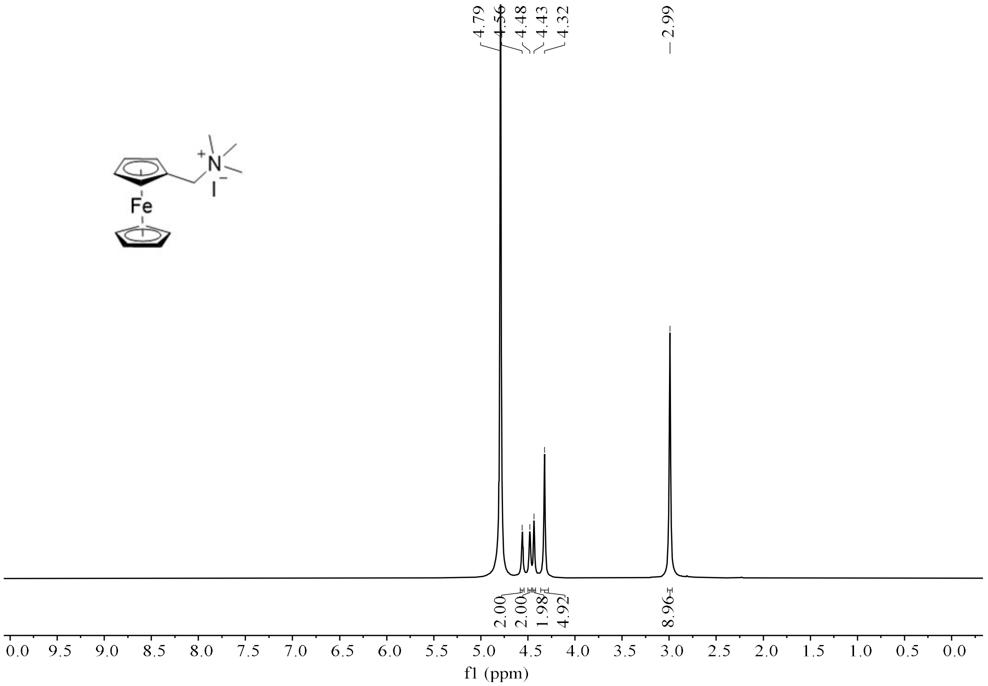

Figure S2 ^{1}H NMR spectrum of S**A3**

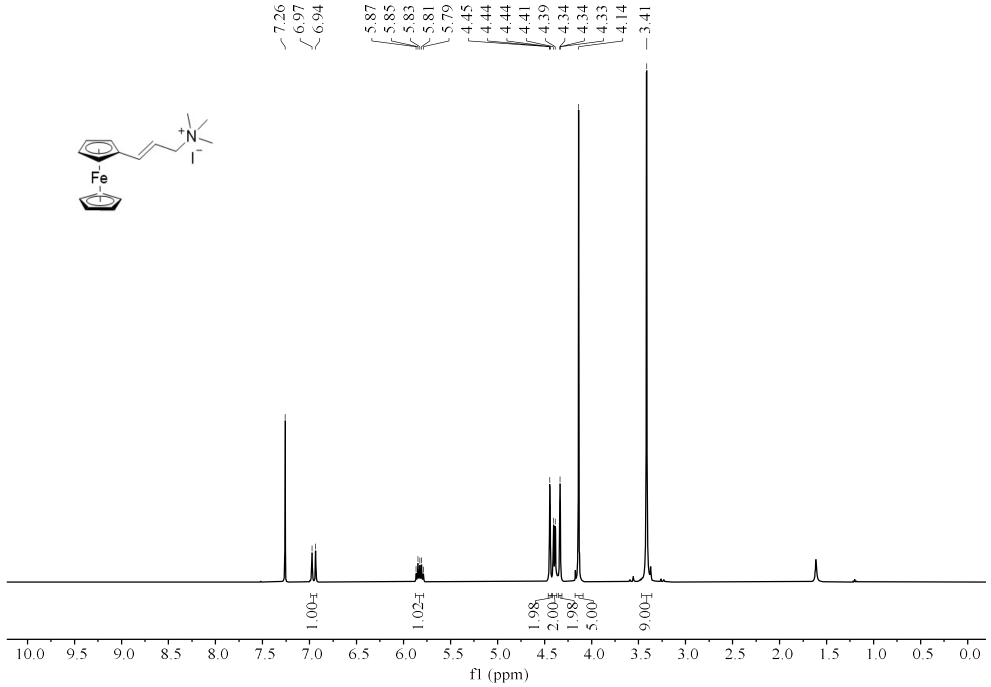

Figure S3 ^{1}H NMR spectrum of S**A5**

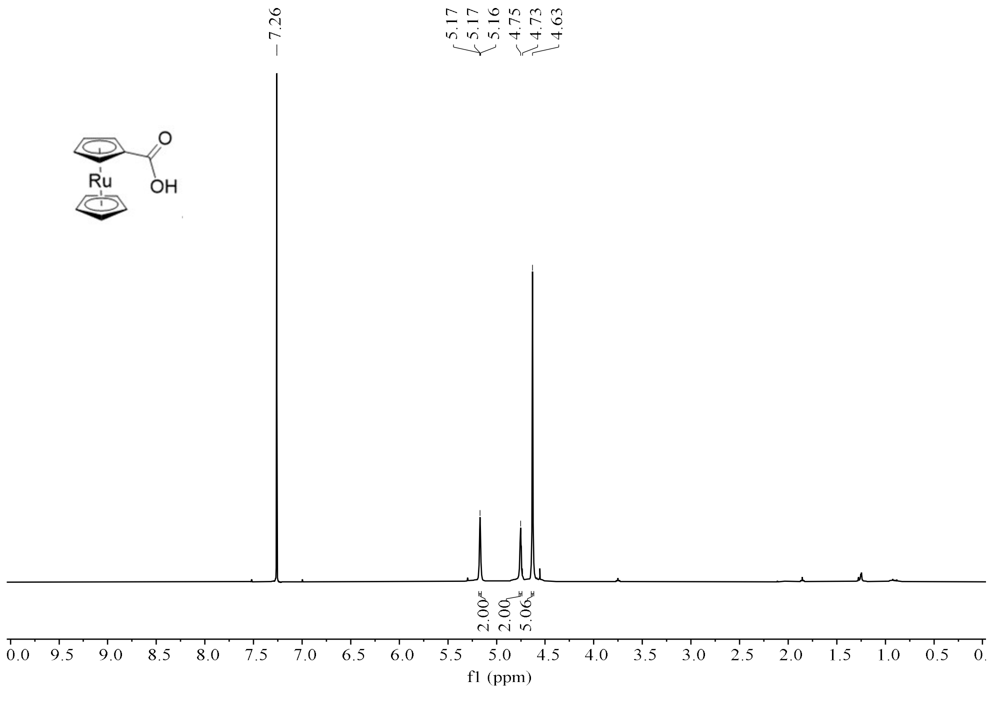

Figure S4 ^{1}H NMR spectrum of **S**B1

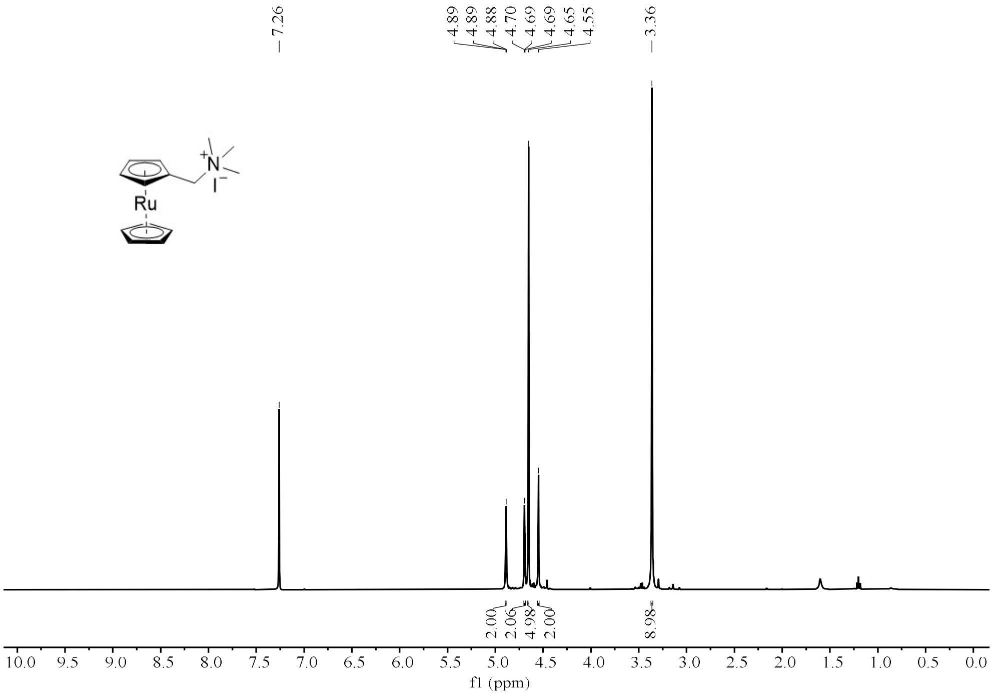

Figure S5 ^{1}H NMR spectrum of **SB2**

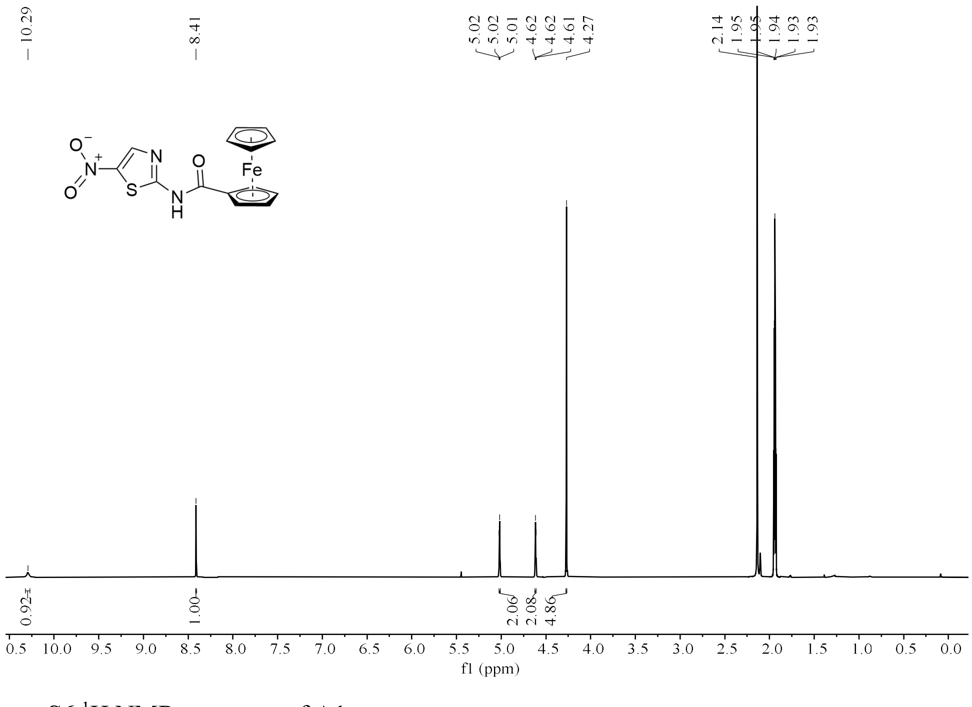

Figure S6 ^{1}H NMR spectrum of **A1**

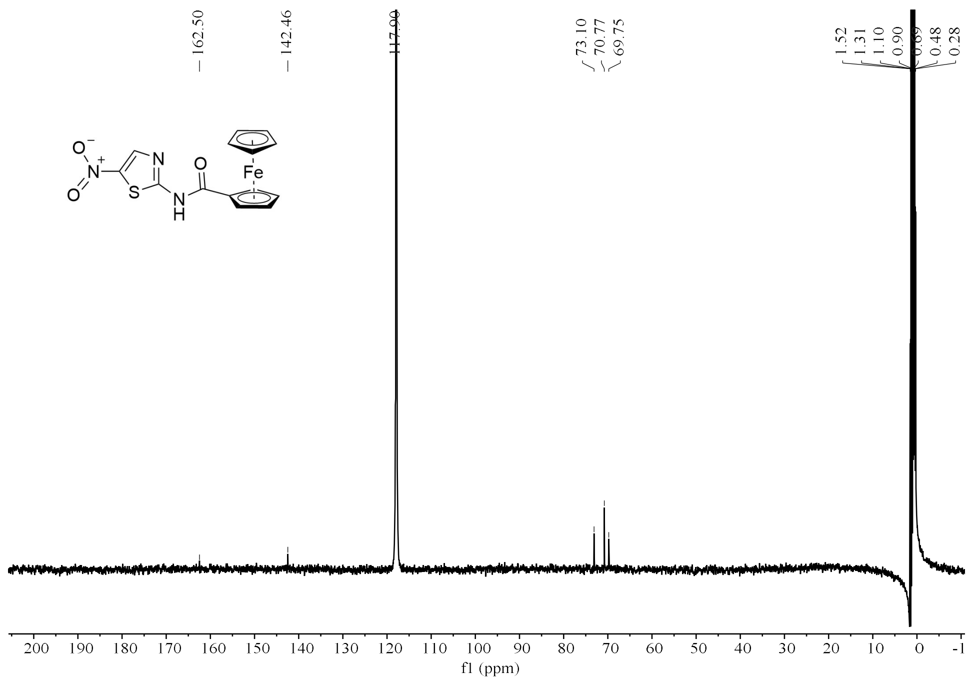

Figure S7 ^{13}C NMR spectrum of **A1**

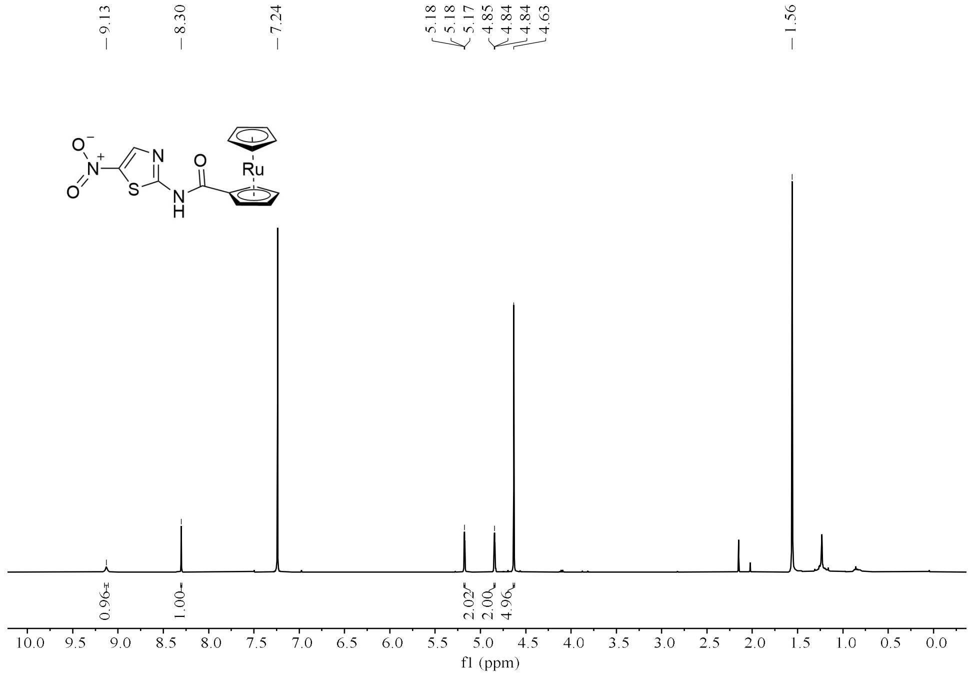

Figure S8 ^{1}H NMR spectrum of **B1**

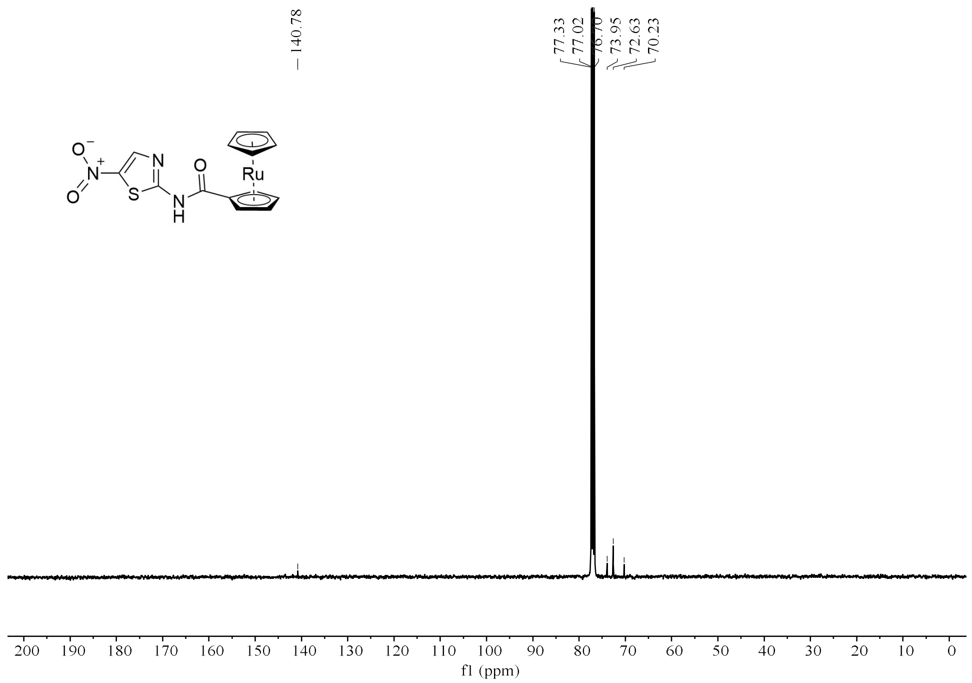

Figure S9 ^{13}C NMR spectrum of **B1**

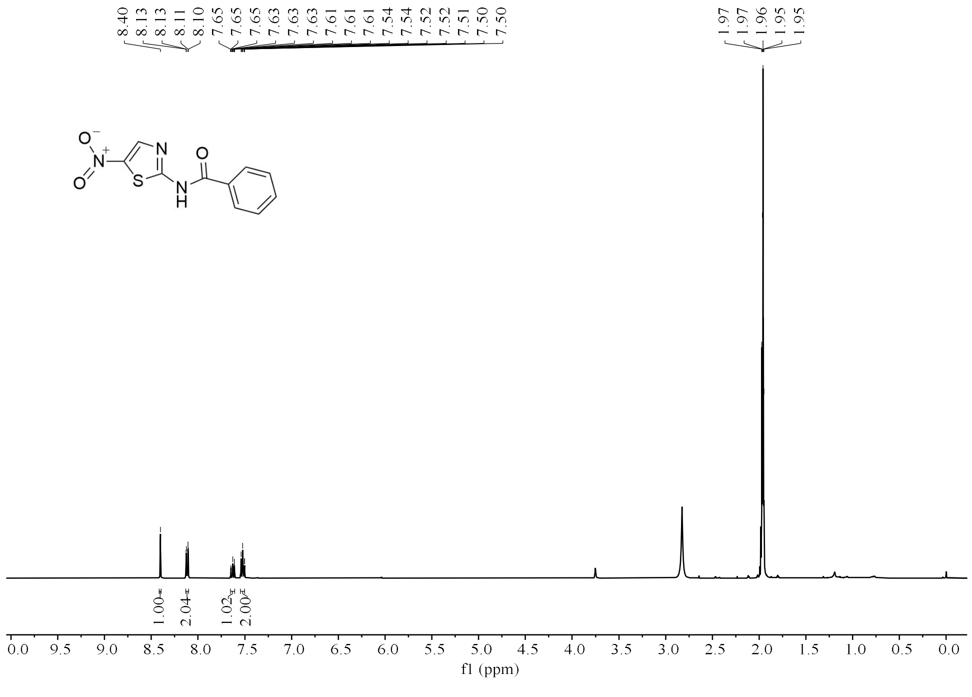

Figure S10 ^{1}H NMR spectrum of **C1**

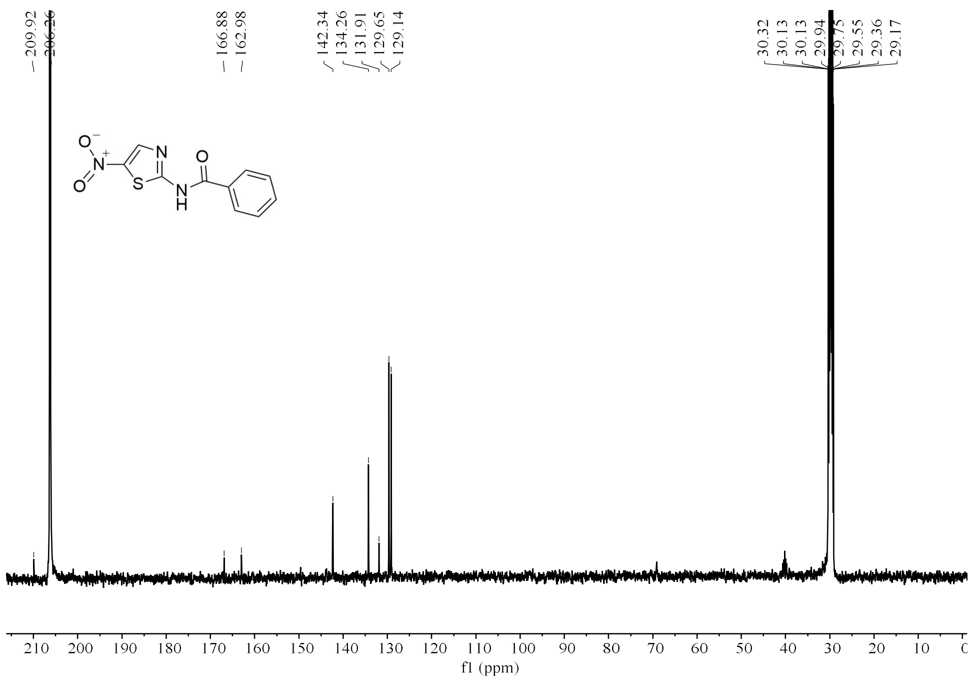

Figure S11 ^{13}C NMR spectrum of **C1**

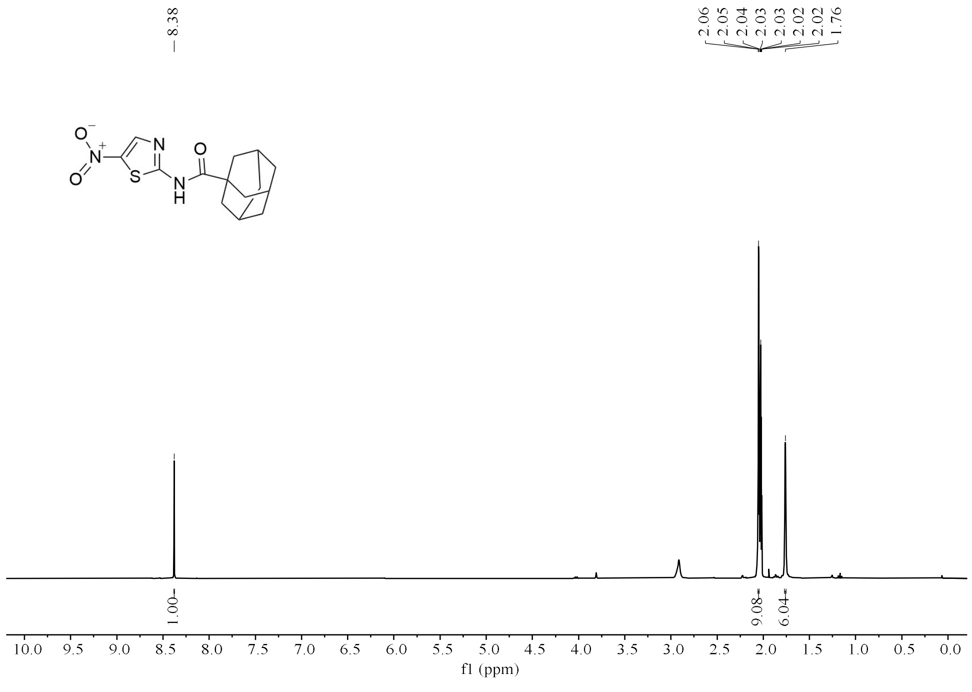

Figure S12 ^{1}H NMR spectrum of **D1**

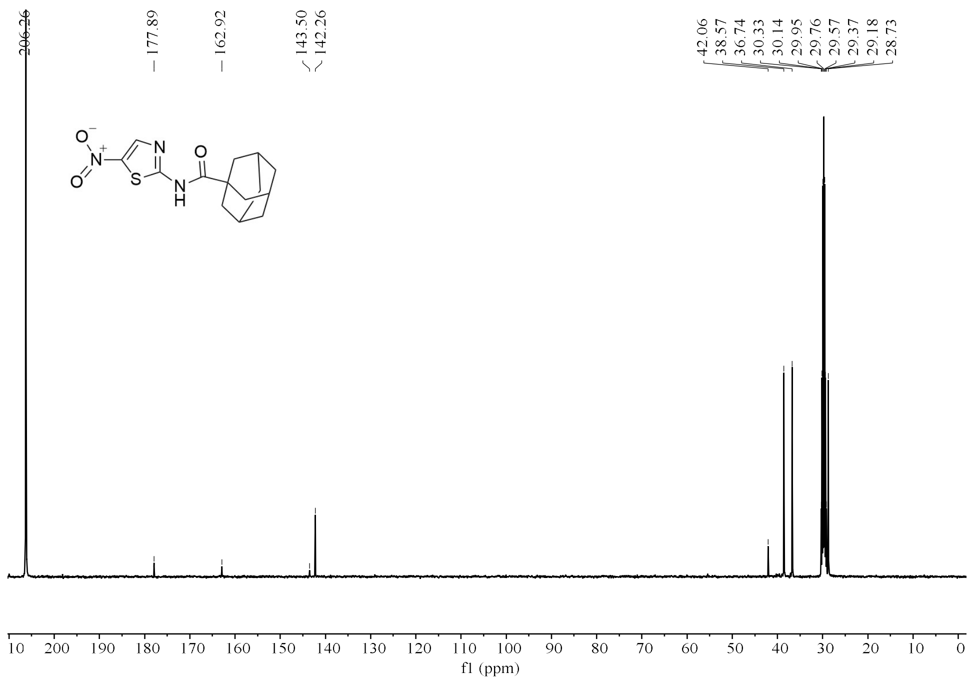

Figure S13 ^{13}C NMR spectrum of **D1**

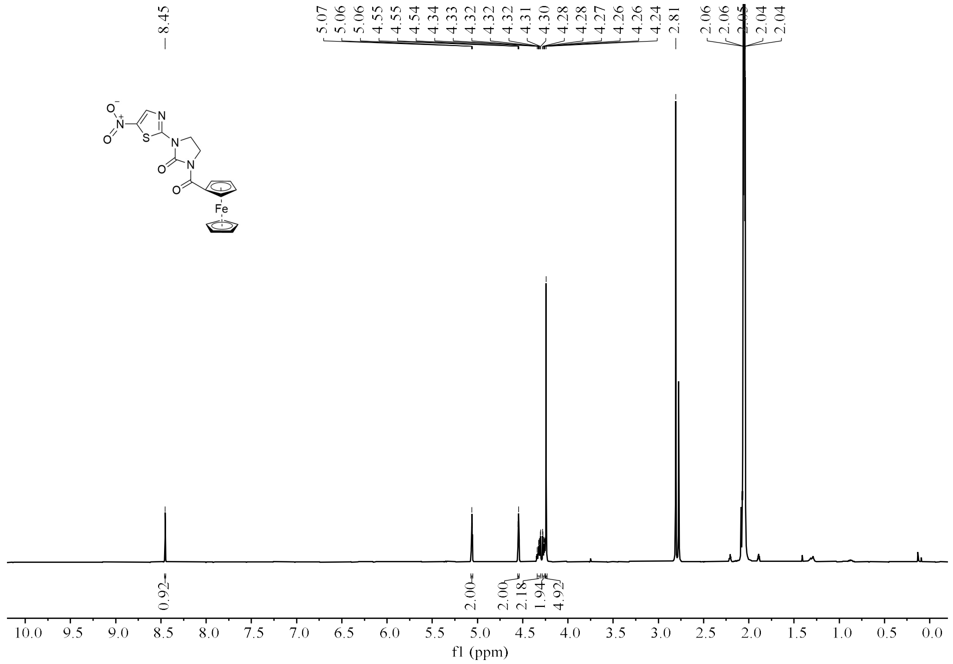

Figure S14 ^{1}H NMR spectrum of **A2**

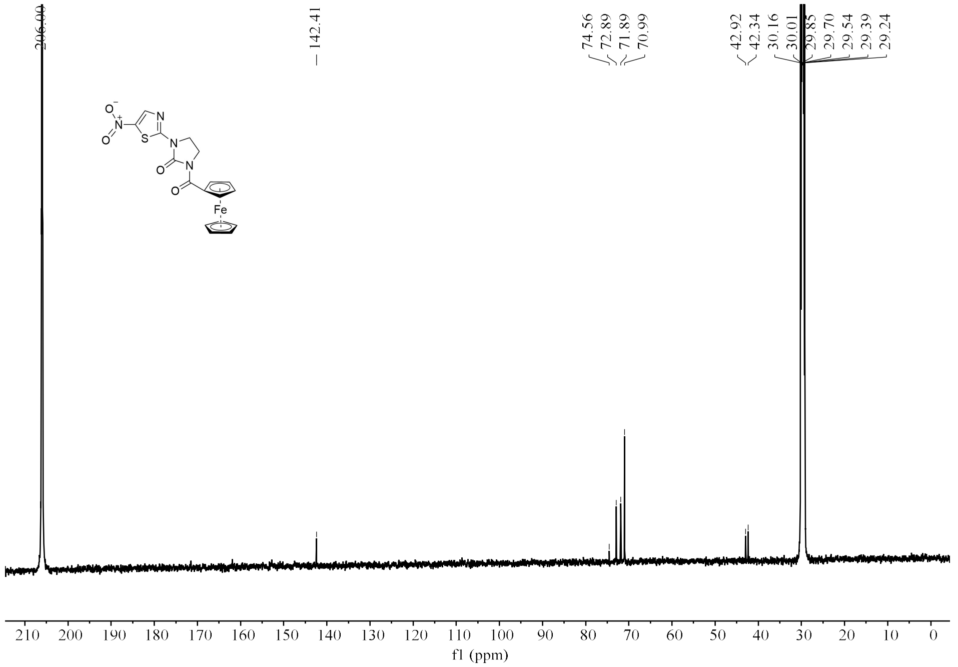

Figure S15 ^{13}C NMR spectrum of **A2**

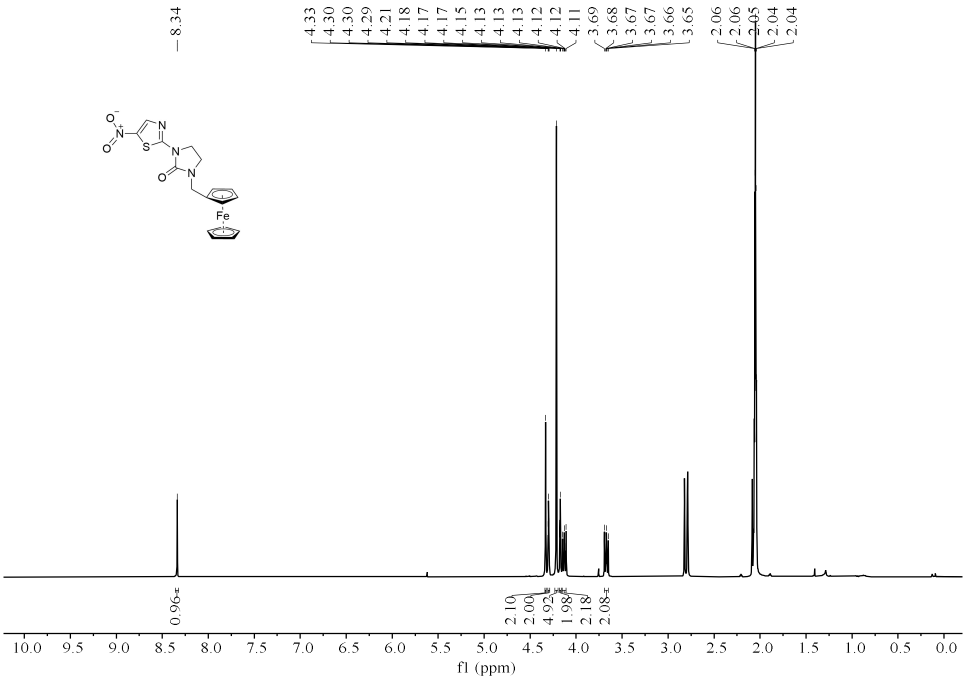

Figure S16 ^{1}H NMR spectrum of **A3**

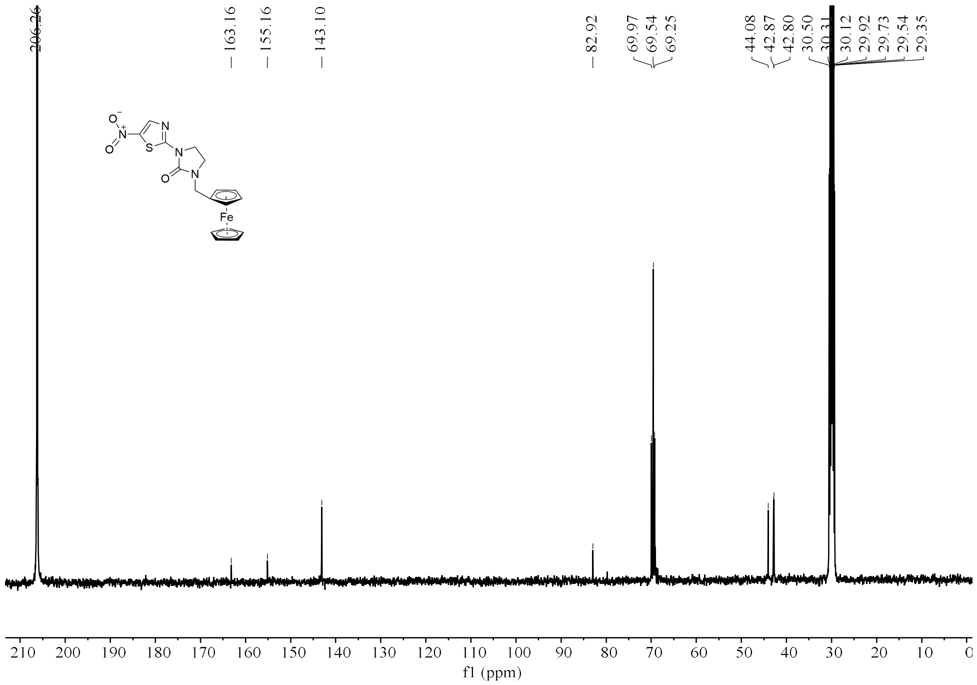

Figure S17 ^{13}C NMR spectrum of **A3**

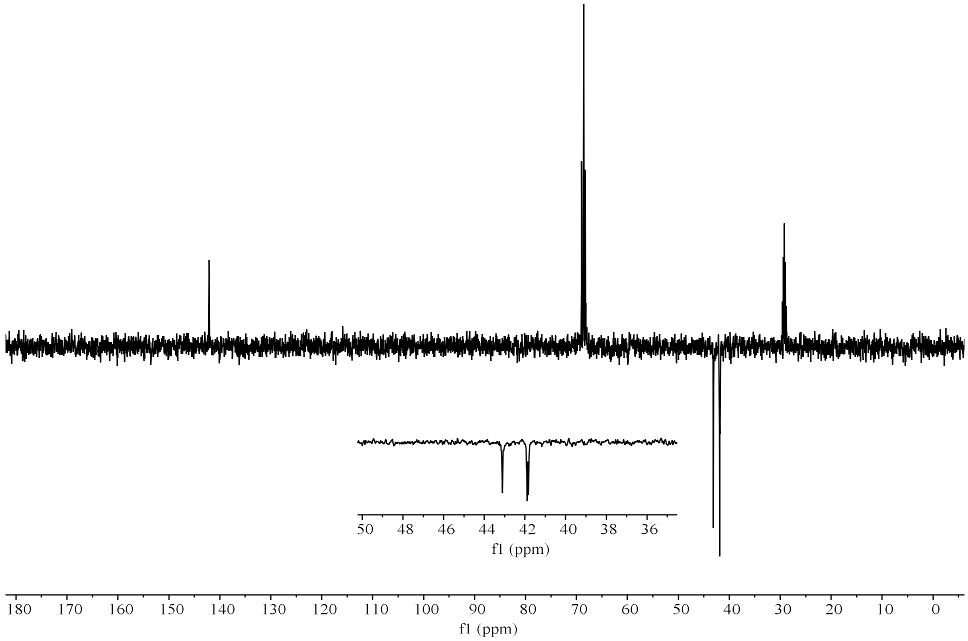

Figure S18 DEPT spectrum of **A3**

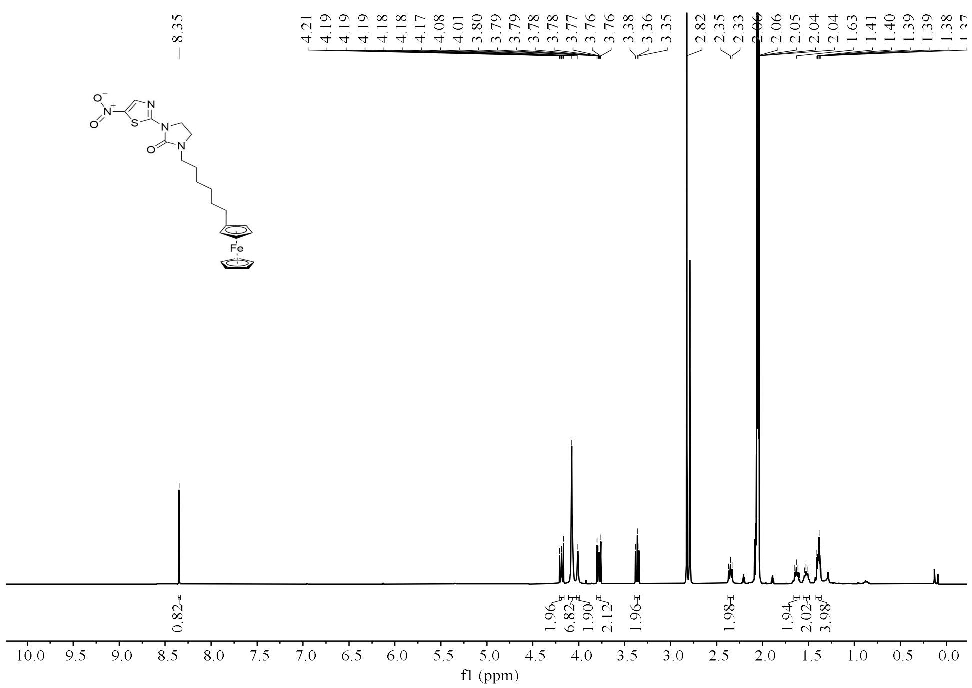

Figure S19 ^{1}H NMR spectrum of **A4**

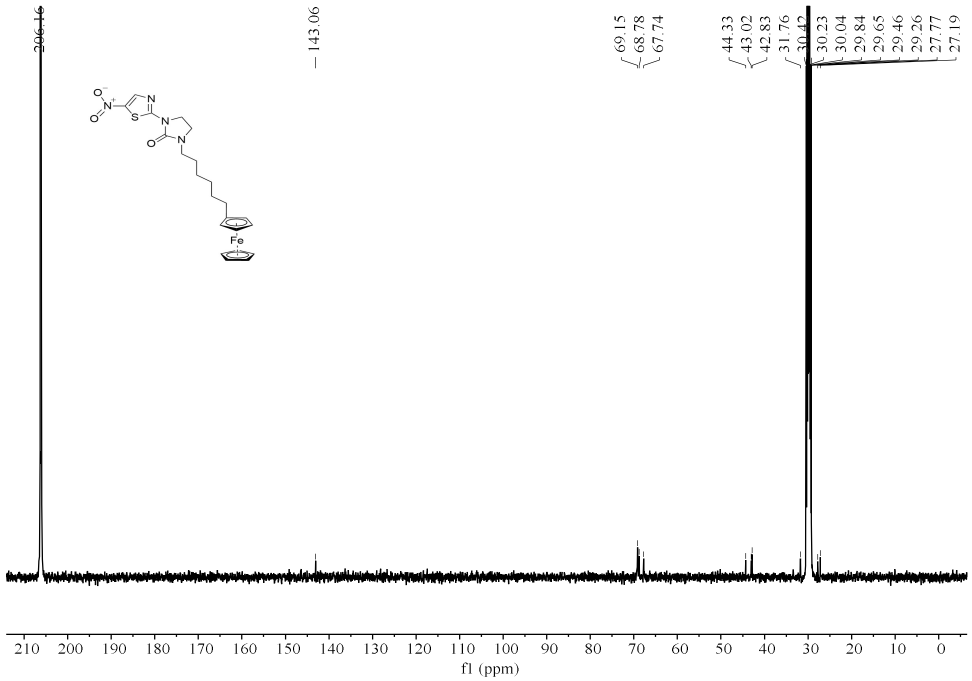

Figure S20 ^{13}C NMR spectrum of **A4**

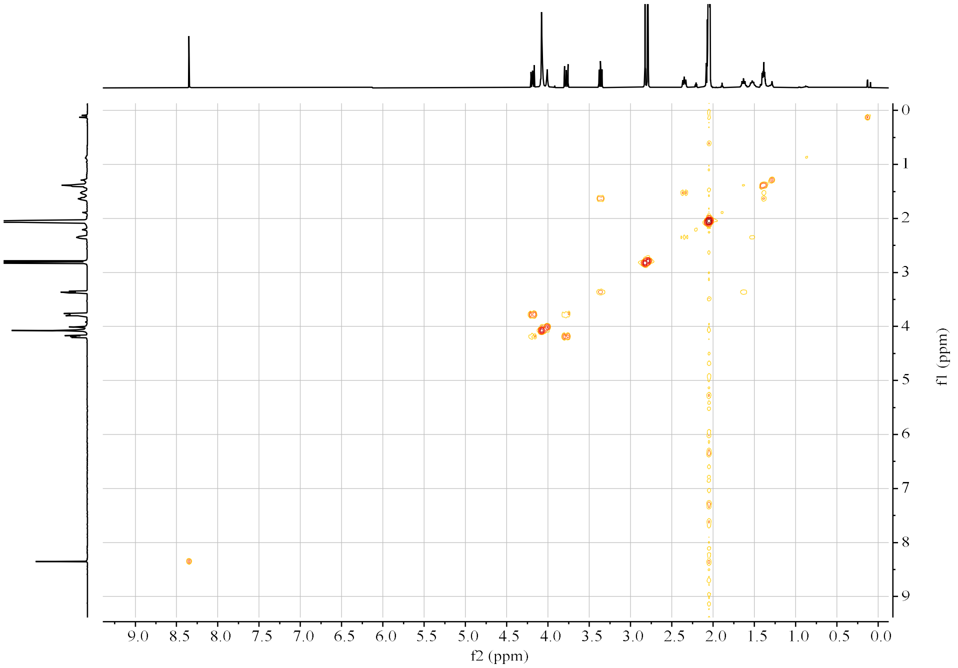

Figure S21 2D-NMR(COSY) spectrum of **A4**

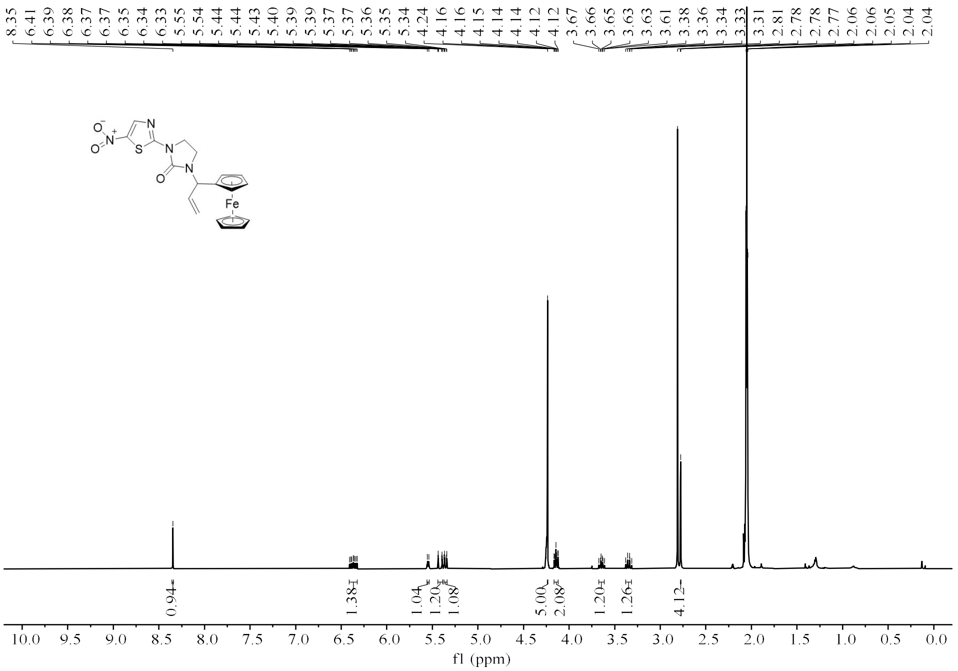

Figure S22 ^{1}H NMR spectrum of **A5**

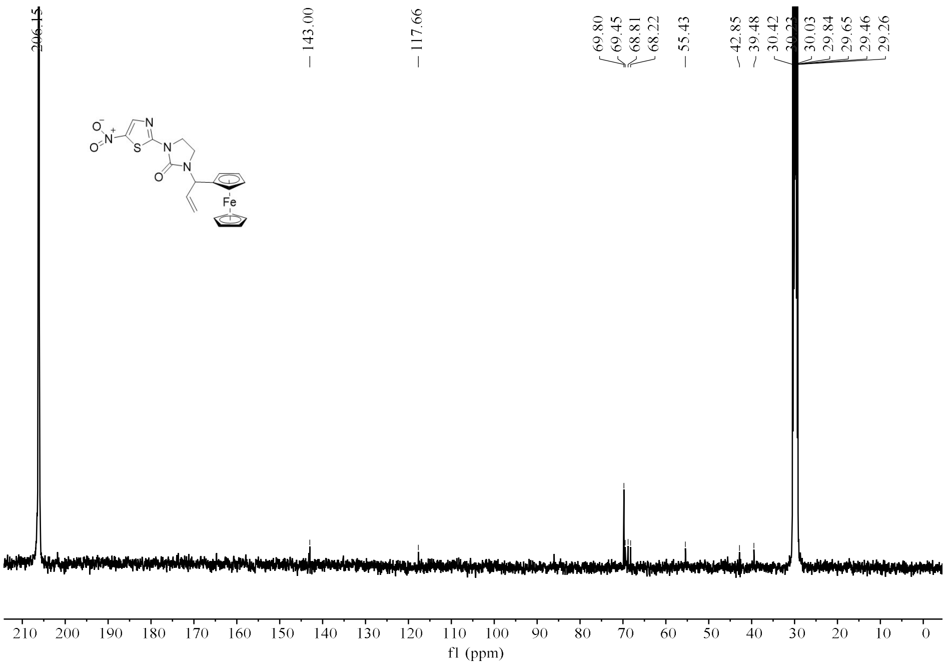

Figure S23 ^{13}C NMR spectrum of **A5**

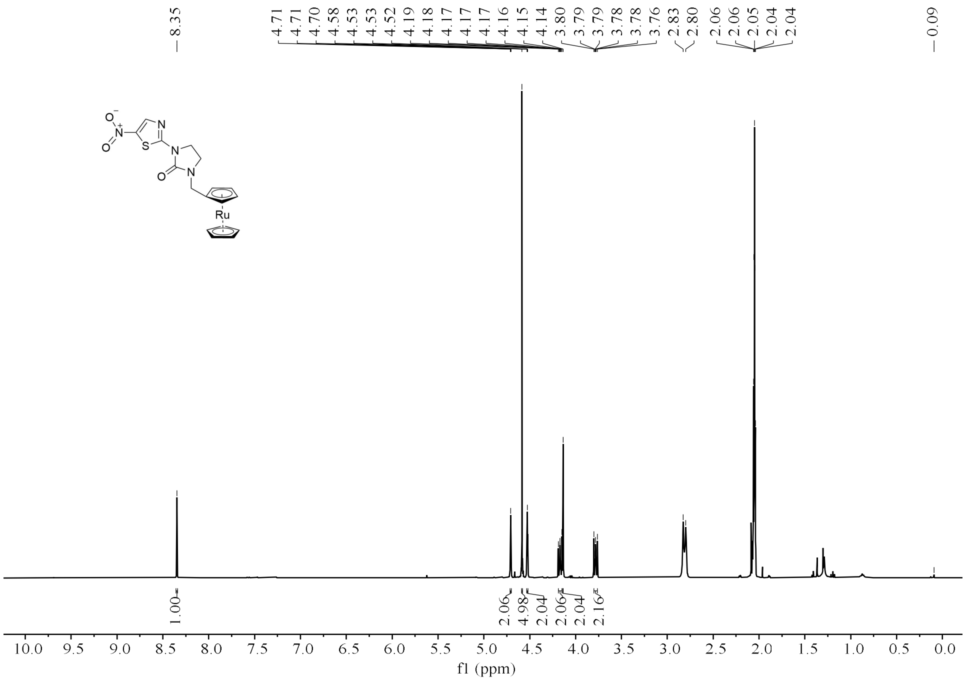

Figure S24 ^{1}H NMR spectrum of **B2**

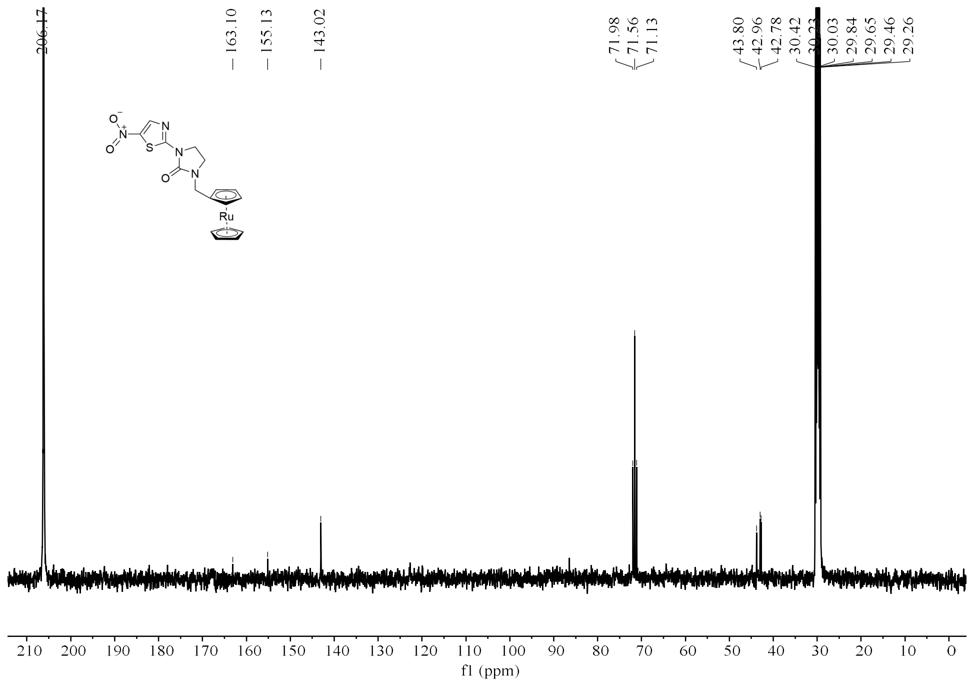

Figure S25 ^{13}C NMR spectrum of **B2**

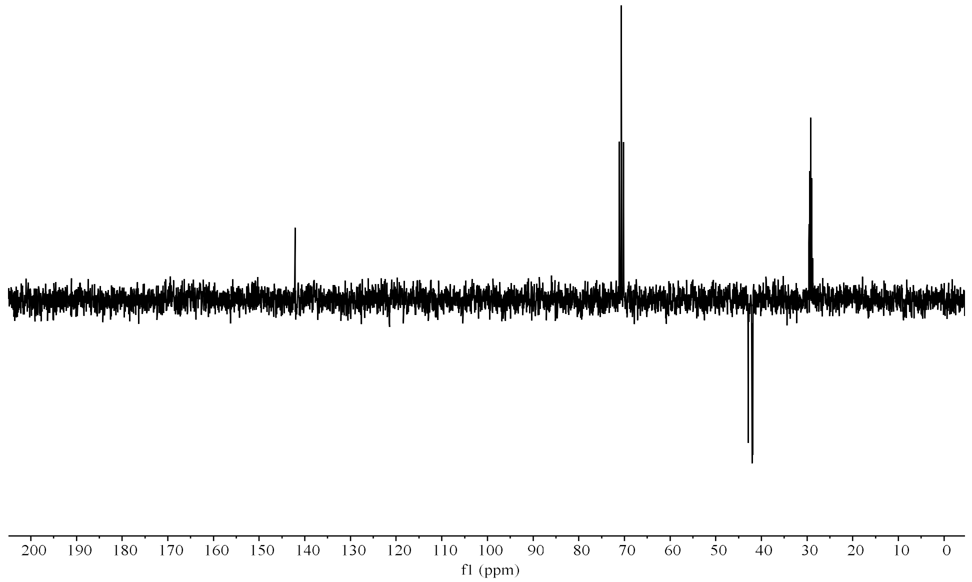

Figure S26 DEPT spectrum of **B2**

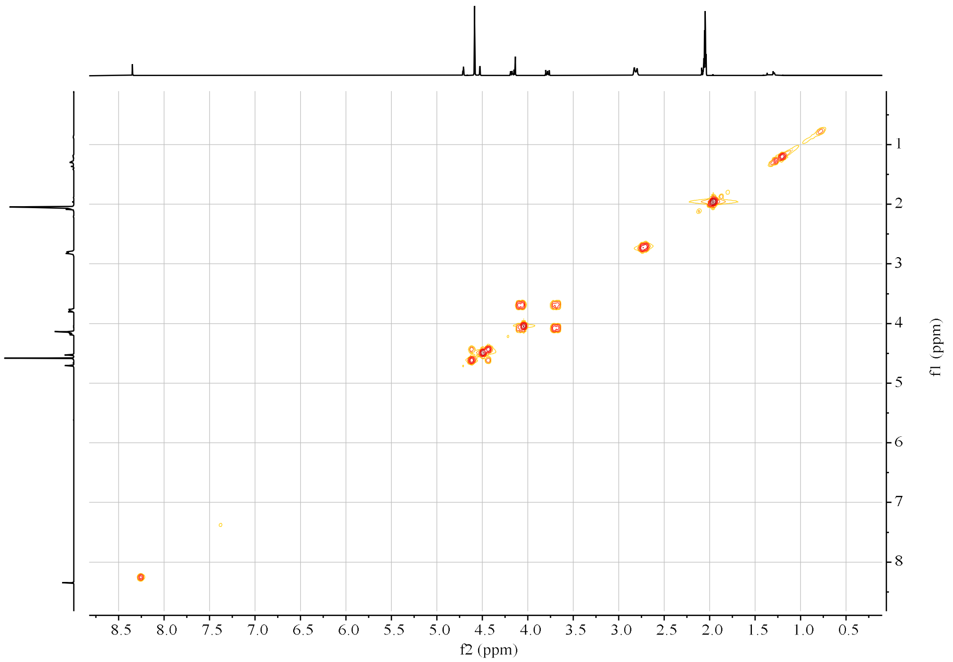

Figure S27 2D-NMR(COSY) spectrum of **B2**

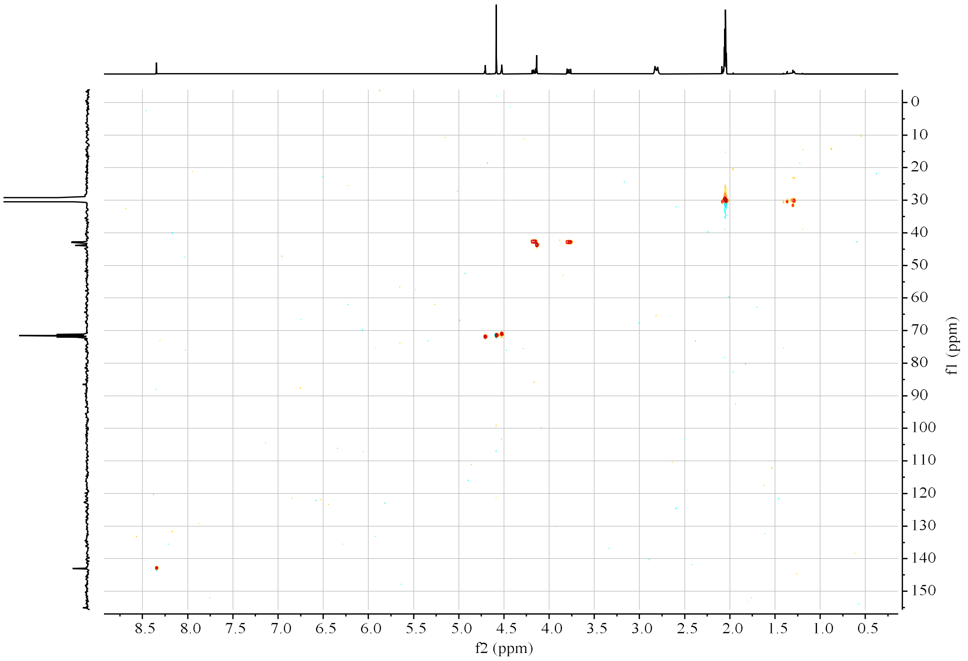

Figure S28 2D-NMR(HSQC) spectrum of **B2**

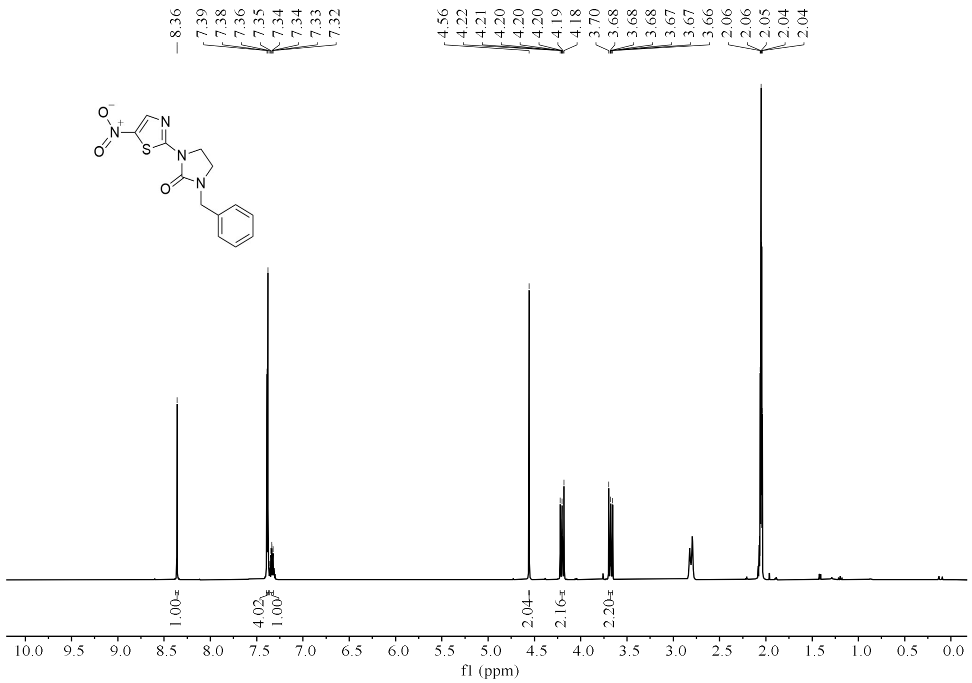

Figure S29 ^{1}H NMR spectrum of **C2**

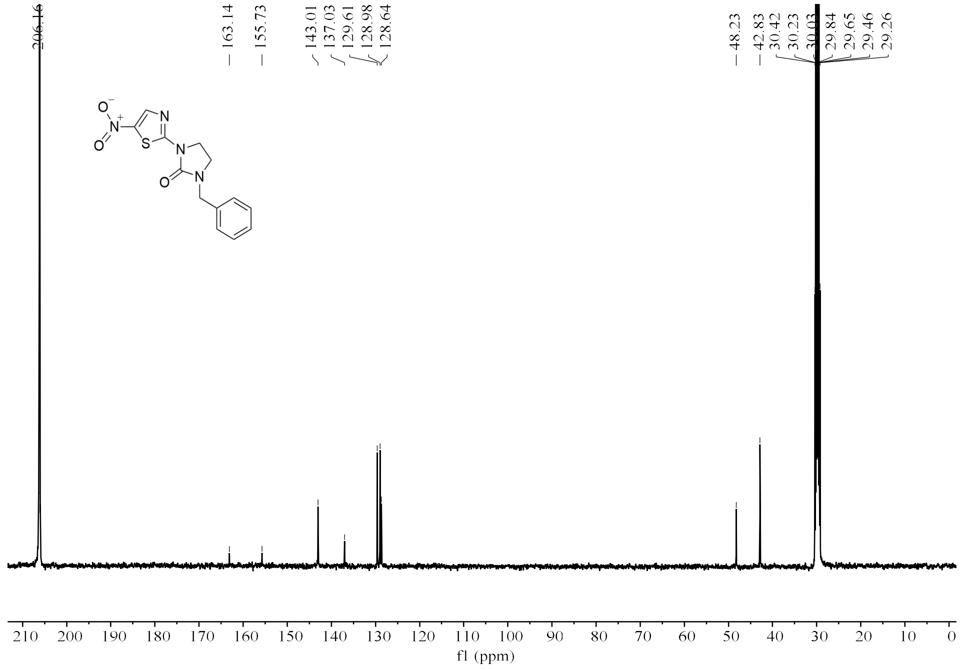

Figure S30 ^{13}C NMR spectrum of **C2**

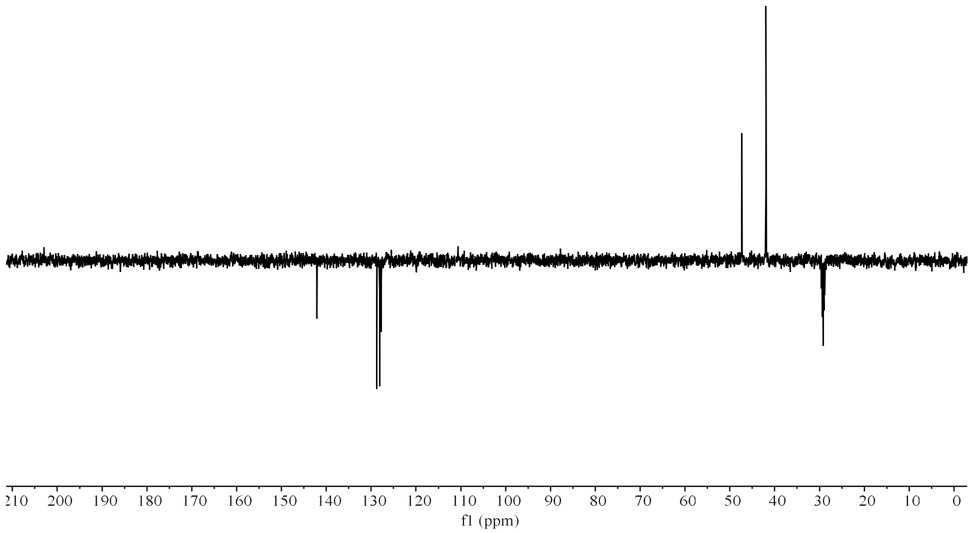

Figure S31 DEPT spectrum of **C2**

4. Stability assays

^{1}H NMR spectra for stability

Preparation of the control samples for ^{1}H NMR analysis: Compounds **B1**, **D1, A3** and **C2** were dissolved in DMSO-d_6. Compound **A2** was dissolved in DMSO-d_6 with D_2O (4:1, v/v). NMR spectra were recorded in deuterated solvents on Bruker Avance-400 in the room temperature for 0, 6, 24, 30 and 48 hours or 0, 3, 6 and 24 hours.

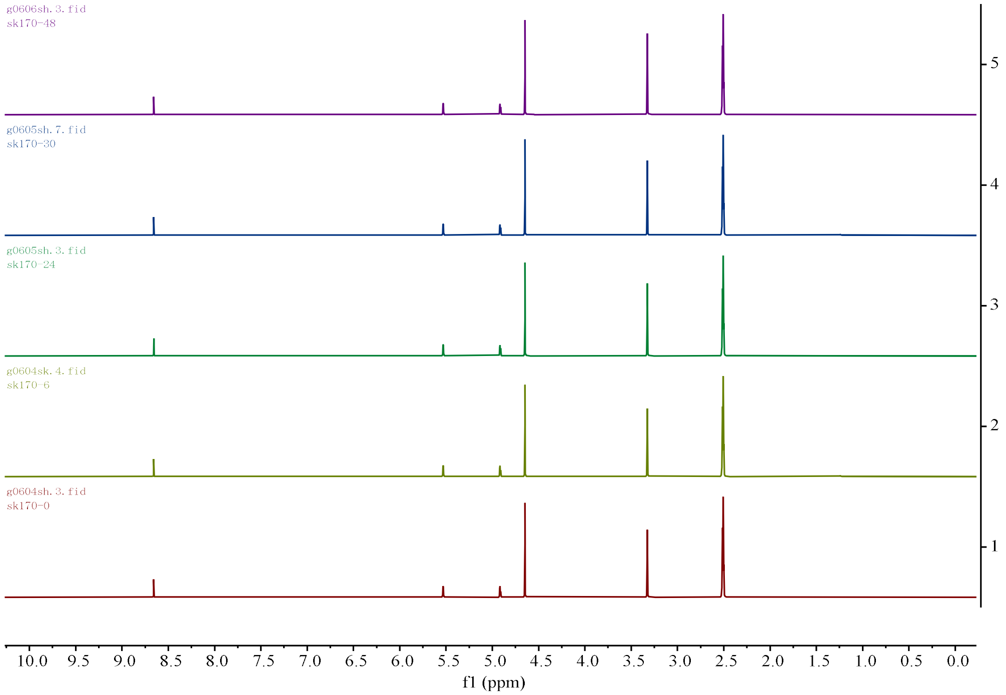

Figure S32 Stability of **B1** in DMSO-d_6 up to two days

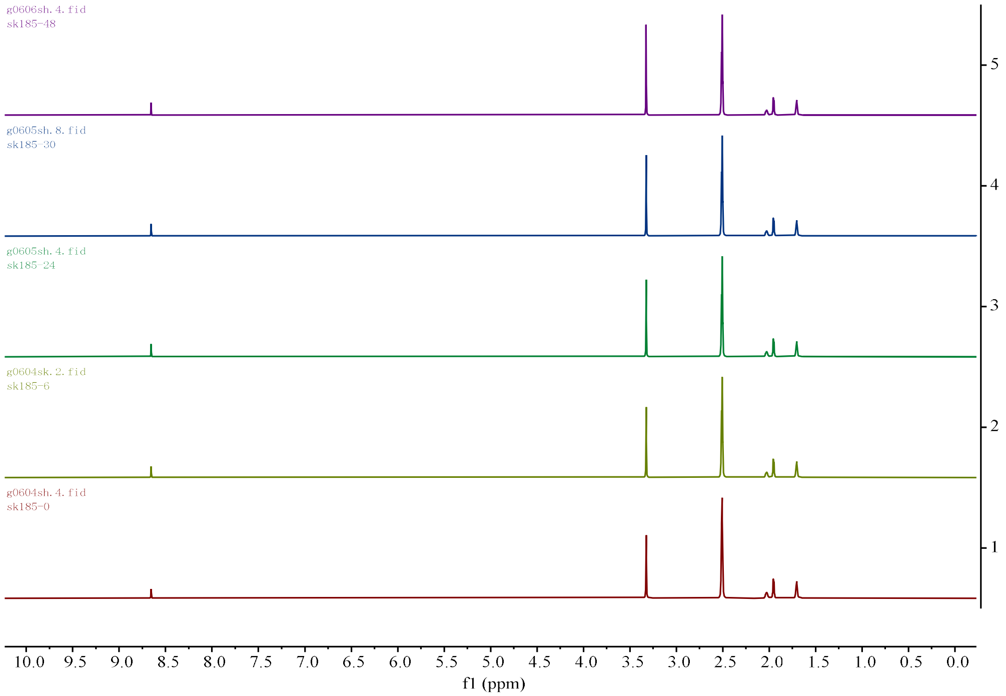

Figure S33 Stability of **D1** in DMSO-d_6 up to two days

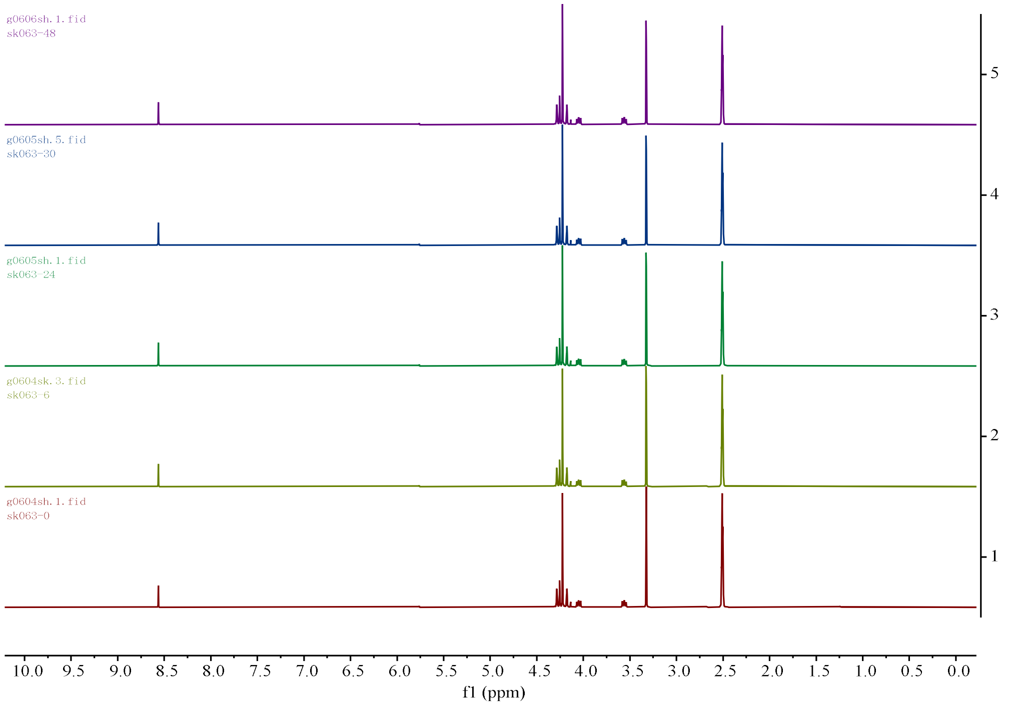

Figure S34 Stability of **A3** in DMSO-d_6 up to two days

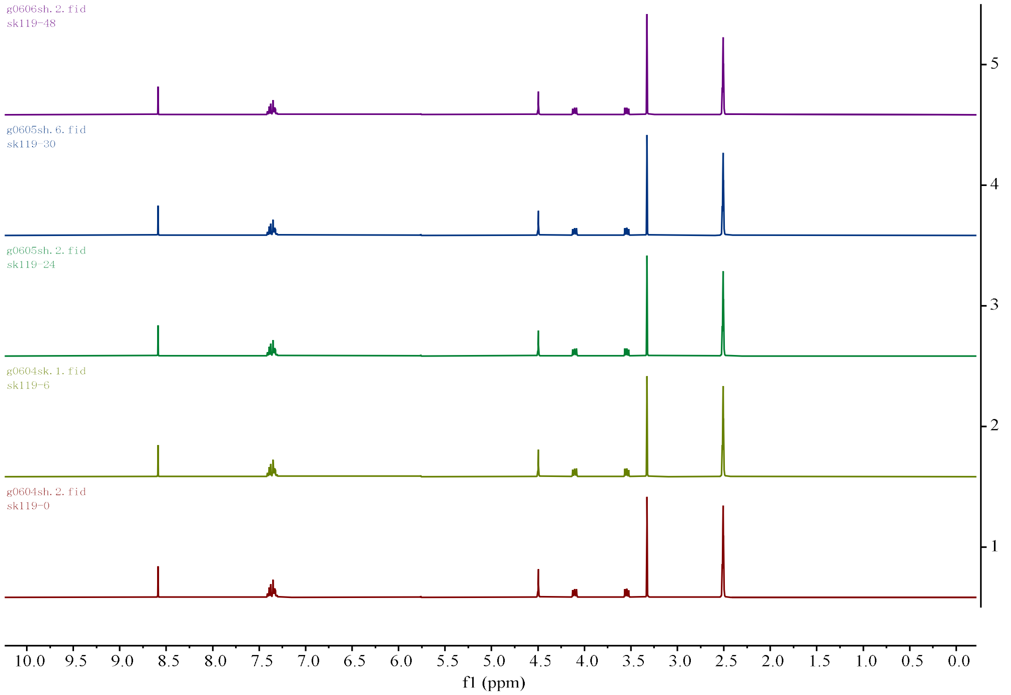

Figure S35 Stability of **C2** in DMSO-d_6 up to two days

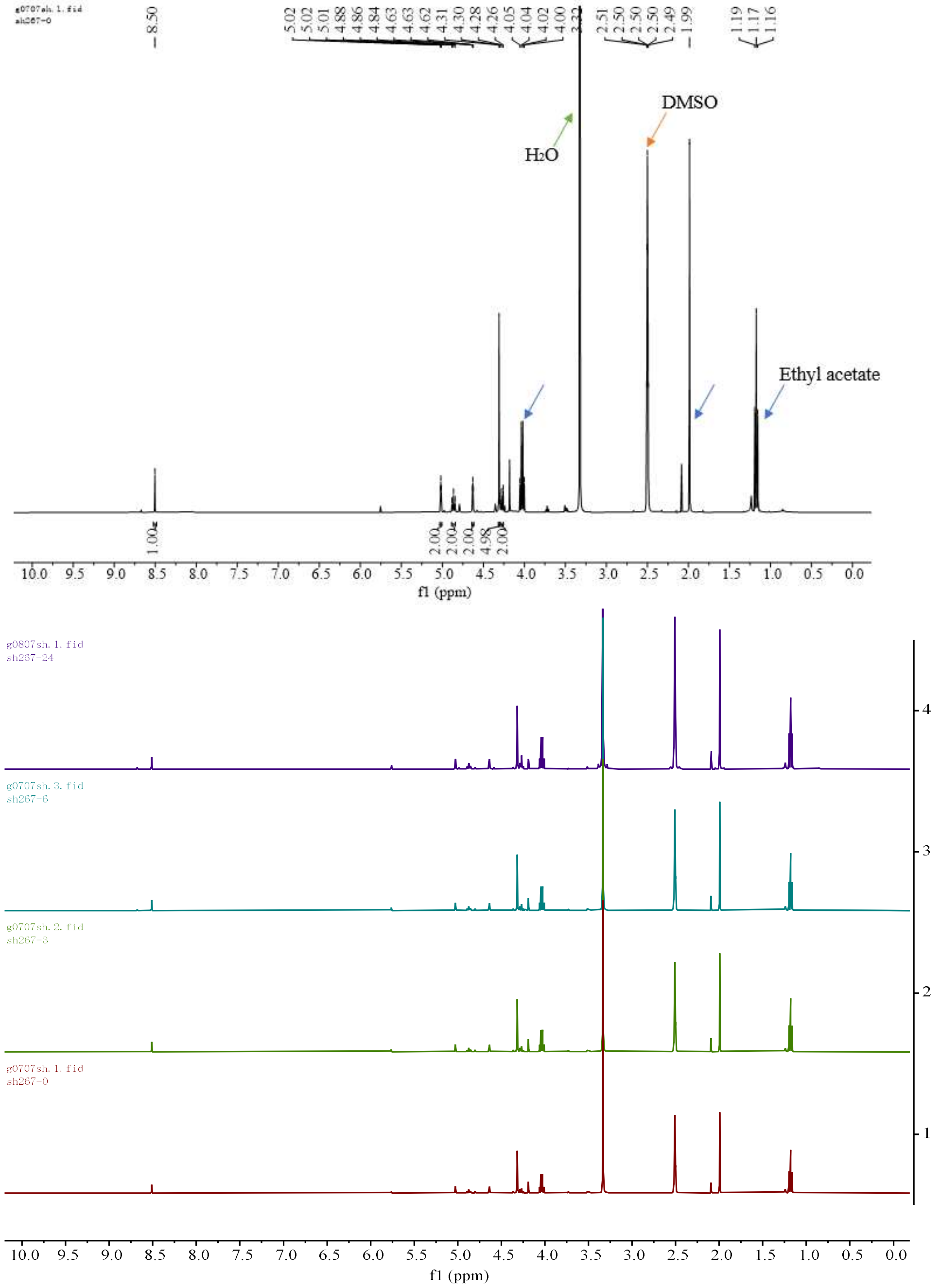

Figure S36 Stability of **A2** in DMSO-d_6 with D_2O up to 24 hours

5. X-ray Crystallography

Single crystal X-ray diffraction data were collected at 160.0(1) K on a Rigaku OD Supernova/Atlas diffractometer for **4**, **7**, **9**, **10** and **12** and on a Rigaku OD Synergy/Pilatus diffractometer for **5** and **11**, using the Cu Kα radiation (λ = 1.54184 Å) from a dual wavelength X-ray source and an Oxford Instruments Cryojet XL cooler. The selected suitable single crystals were mounted using polybutene oil on a flexible

loop fixed on a goniometer head and immediately transferred to the diffractometer. Pre-experiments, data collections, data reductions and analytical absorption corrections[16] were performed with the program suite *CrysAlisPro*.[17] Using *Olex*2,[18] the structures were solved with the SHELXT[19] small molecule structure solution program and refined with the *SHELXL2018/3* program package[20] by full-matrix least-squares minimization on F^2. *PLATON*[21] was used to validate the result of the X-ray analyses. Crystal data, data collection and structure refinement details are summarized in Tables S4-S7. CCDC-2465652 (**4**), CCDC-2465653 (**5**), CCDC-2465654 (**7**), CCDC-2465655 (**9**), CCDC-2465656 (**10**), CCDC-2465657 (**11**) and CCDC-2465658 (**12**) contain the supplementary crystallographic data for this paper. The data can be obtained free of charge from The Cambridge Crystallographic Data Centre via www.ccdc.cam.ac.uk/structures.

In the crystal structure of **4**, there are two independent molecules of the main compound $C_{14}H_{17}N_3O_3S$ and one solvent molecule of water. The H-atoms attached to the N-atoms were located in a different Fourier map and freely refined (positions and isotropic displacement parameters). The H-atoms of the isolated water molecule were located in a different Fourier map, placed and refined with the DFIX 0.87 0.02 instruction, in association with $U_{iso}(H) = 1.5U_{eq}(O)$. All remaining H-atoms were placed geometrically and refined isotropically using a riding model, with C-H = 0.95 Å (C-aromatic), 0.99 Å (C-methylene) and 1.00 Å (C-methine), in association with $U_{iso}(H) = 1.2U_{eq}(C)$. The crystal structure of compound **5** has been solved and refined successfully with no unusual features. The H-atom attached to the N-atom was located in a different Fourier map and freely refined (position and isotropic displacement parameter). All remaining H-atoms were placed geometrically and refined isotropically using a riding model, with C-H = 0.95 Å and $U_{iso}(H) = 1.2U_{eq}(C)$. The crystal structure of compound **7** has been solved and refined successfully with no unusual features. All H-atoms were placed geometrically and refined isotropically using a riding model, with C-H = 0.95 Å (C-aromatic) and 0.99 Å (C-methylene), in association with $U_{iso}(H) = 1.2U_{eq}(C)$. In the crystal structure of compound **9**, solvent molecules of acetone

cocrystallized with the main species in a ratio 2/1, respectively. The molecules of acetone are fully disordered over two sets of positions with a site-occupancy factor of 0.5; the positions are near center of inversions. Restraints were applied in *SHELXL* to the bond distances in the disordered parts using the *SADI* and DFIX instructions, and to the atomic displacement parameters of the disordered atoms using the *SIMU* instruction. All H-atoms were placed geometrically and refined isotropically using a riding model, with C-H = 0.95 Å (C-aromatic), 0.98 Å (C-methyl), and 0.99 Å (C-methylene), in association with U_{iso}(H) = 1.2U_{eq}(C) or 1.5U_{eq}(C-methyl). In the crystal structure of compound **10**, the ferrocene (except the metal center) and three CH_2 groups of the C_6 chain are disordered over two sets of positions (named A and B) with site-occupancy factors of 0.494(2) and 0.506(2). Restraints were applied in *SHELXL* to the bond distances in the disordered parts using the *SADI* instruction, and to the atomic displacement parameters of the disordered atoms using the *SIMU* instruction. All H-atoms were placed geometrically and refined isotropically using a riding model, with C-H = 0.95 Å (C-aromatic) and 0.99 Å (C-methylene), in association with U_{iso}(H) = 1.2U_{eq}(C). The crystal structure of compound **11** exhibits two independent molecules in the asymmetric unit. The molecules are chiral (chiral carbon atoms C11 and C30), they crystallized in a centrosymmetric space group as a racemic mixture. All H-atoms were placed geometrically and refined isotropically using a riding model, with C-H = 0.95 Å (C-aromatic), 0.99 Å (C-methylene) and 1.00 Å (C-methine), in association with U_{iso}(H) = 1.2U_{eq}(C). The crystal structure of compound **12** was refined as a non-merohedral 2-component twin. The twin was identified and indexed using the Ewald Explorer tool of *CrysAlisPro*.[17] The minor component is rotated by -179.96 degrees around vector (0.00 0.00 1.00) in the reciprocal space (hkl) and around vector (0.38 0.00 0.93) in the direct space (uvw). Two data sets were produced: an HKLF-4 data set used to solve the structure, and an HKLF-5 data set used for the final refinements. The scale factor of the major component is 0.8837(14). All H-atoms were placed geometrically and refined isotropically using a riding model, with C-H = 0.95 Å (C-aromatic) and 0.99 Å (C-methylene), in association with U_{iso}(H) = 1.2U_{eq}(C).

Table S4 Selected crystal data and structure refinement parameters for **A1** and **A2**.

CCDC Number	CCDC-2465653	CCDC-2465658
Empirical formula	$C_{14}H_{11}FeN_3O_3S$	$C_{17}H_{14}FeN_4O_4S$
Formula weight	357.17	426.23
Temperature/K	160(1)	160(1)
Crystal system	orthorhombic	monoclinic
Space group	Pbcn	$P2_1/c$
a/Å	17.9469(2)	6.3036(2)
b/Å	10.18830(10)	7.7424(2)
c/Å	14.8740(2)	32.7850(8)
α/	90	90
β/	90	94.468(2)
γ/	90	90
Volume/Å	2719.69(5)	1595.21(8)
Z	8	4
ρ_{calc}g/cm^3	1.745	1.775
μ/mm^{-1}	10.491	9.131
F(000)	1456.0	872.0
Crystal size/mm^3	0.12 × 0.11 × 0.02	0.19 × 0.04 × 0.04
Radiation	Cu Kα (λ = 1.54184)	Cu Kα (λ = 1.54184)
2Θ range for data collection/	9.856 to 149.006	5.408 to 153.892
Index ranges	-19 ≤ h ≤ 22, -11 ≤ k ≤ 12, -18 ≤ l ≤ 18	-7 ≤ h ≤ 7, -9 ≤ k ≤ 9, -41 ≤ l ≤ 39
Reflections collected	15412	15518
Independent reflections	2788 [Rint = 0.0280, Rsigma = 0.0206]	15518 [Rint = ?, Rsigma = 0.0423]
Data/restraints/parameters	2788/0/203	15518/0/245
Goodness-of-fit on F^2	1.071	1.037
Final R indexes [I>=2σ (I)]	R1 = 0.0253, wR2 = 0.0682	R1 = 0.0510, wR2 = 0.1368
Final R indexes [all data]	R1 = 0.0272, wR2 = 0.0695	R1 = 0.0575, wR2 = 0.1413
Largest diff. peak/hole / e Å^{-3}	0.28/-0.34	0.54/-0.27

Table S5 Selected crystal data and structure refinement parameters for **A4** and **A5**.

CCDC Number	CCDC-2465656	CCDC-2465657
Empirical formula	$C_{22}H_{26}FeN_4O_3S$	$C_{19}H_{18}FeN_4O_3S$
Formula weight	482.38	438.28
Temperature/K	160(1)	160(1)
Crystal system	triclinic	monoclinic
Space group	P-1	P21/n
a/Å	6.36820(10)	14.11265(10)
b/Å	10.3531(2)	11.32848(10)
c/Å	16.2843(3)	23.07869(18)
α/	95.757(2)	90
β/	98.699(2)	96.3137(7)
γ/	97.243(2)	90
Volume/Å	1044.98(3)	3667.32(5)
Z	2	8
$\rho_{calc}g/cm^3$	1.533	1.588
μ/mm^{-1}	6.999	7.917
F(000)	504.0	1808.0
Crystal size/mm^3	0.19 × 0.07 × 0.04	0.15 × 0.12 × 0.08
Radiation	Cu Kα (λ = 1.54184)	Cu Kα (λ = 1.54184)
2Θ range for data collection/	5.532 to 148.962	7.016 to 148.996
Index ranges	-7 ≤ h ≤ 7, -12 ≤ k ≤ 12, -20 ≤ l ≤ 19	-17 ≤ h ≤ 17, -13 ≤ k ≤ 14, -28 ≤ l ≤ 25
Reflections collected	19043	39976
Independent reflections	4235 [Rint = 0.0341, Rsigma = 0.0244]	7483 [Rint = 0.0284, Rsigma = 0.0211]
Data/restraints/parameters	4235/656/398	7483/60/505
Goodness-of-fit on F^2	1.065	1.039
Final R indexes [I>=2σ (I)]	R1 = 0.0387, wR2 = 0.0990	R1 = 0.0279, wR2 = 0.0706
Final R indexes [all data]	R1 = 0.0415, wR2 = 0.1015	R1 = 0.0312, wR2 = 0.0723
Largest diff. peak/hole / e $Å^{-3}$	0.67/-0.41	0.58/-0.36

Table S6 Selected crystal data and structure refinement parameters for **B2** and **C2**.

CCDC Number	CCDC-2465655	CCDC-2465654
Empirical formula	$C_{37}H_{38}N_8O_7Ru_2S_2$	$C_{13}H_{12}N_4O_3S$
Formula weight	973.01	304.33
Temperature/K	160(1)	160(1)
Crystal system	monoclinic	monoclinic
Space group	$P2_1/n$	C2/c
a/Å	6.25922(6)	26.4923(5)
b/Å	13.79109(13)	7.7708(2)
c/Å	22.10292(19)	12.8391(2)
α/	90	90
β/	90.2672(8)	91.949(2)
γ/	90	90
Volume/Å	1907.93(3)	2641.61(9)
Z	2	8
$\rho_{calc}g/cm^3$	1.694	1.530
μ/mm^{-1}	7.946	2.347
F(000)	984.0	1264.0
Crystal size/mm^3	0.18 × 0.07 × 0.04	0.17 × 0.13 × 0.06
Radiation	Cu Kα (λ = 1.54184)	Cu Kα (λ = 1.54184)
2Θ range for data collection/	7.556 to 149.006	6.676 to 148.994
Index ranges	-7 ≤ h ≤ 7, -15 ≤ k ≤ 17, -27 ≤ l ≤ 26	-32 ≤ h ≤ 32, -9 ≤ k ≤ 9, -14 ≤ l ≤ 16
Reflections collected	19755	12272
Independent reflections	3889 [Rint = 0.0184, Rsigma = 0.0105]	2692 [R_{int} = 0.0227, R_{sigma} = 0.0129]
Data/restraints/parameters	3889/21/273	2692/0/190
Goodness-of-fit on F^2	1.057	1.082
Final R indexes [I>=2σ (I)]	R1 = 0.0188, wR2 = 0.0473	R_1 = 0.0343, wR_2 = 0.0967
Final R indexes [all data]	R1 = 0.0191, wR2 = 0.0475	R_1 = 0.0350, wR_2 = 0.0979
Largest diff. peak/hole / e $Å^{-3}$	0.42/-0.66	0.61/-0.90

Table S7 Selected crystal data and structure refinement parameters for **D1**.

CCDC Number	CCDC-2465652
Empirical formula	$C_{28}H_{36}N_6O_7S_2$
Formula weight	632.75
Temperature/K	160(1)
Crystal system	monoclinic
Space group	$P2_1/c$
a/Å	18.31710(15)
b/Å	13.47448(10)
c/Å	12.21859(10)
α/	90
β/	106.3121(9)
γ/	90
Volume/Å	2894.32(4)
Z	4
ρ_{calc}g/cm^3	1.452
μ/mm^{-1}	2.162
F(000)	1336.0
Crystal size/mm^3	0.2 × 0.1 × 0.05
Radiation	Cu Kα (λ = 1.54184)
2Θ range for data collection/	5.026 to 152.882
Index ranges	-23 ≤ h ≤ 22, -16 ≤ k ≤ 16, -15 ≤ l ≤ 13
Reflections collected	29969
Independent reflections	5992 [R_{int} = 0.0164, R_{sigma} = 0.0117]
Data/restraints/parameters	5992/3/402
Goodness-of-fit on F^2	1.042
Final R indexes [I>=2σ (I)]	R_1 = 0.0285, wR_2 = 0.0771
Final R indexes [all data]	R_1 = 0.0304, wR_2 = 0.0788
Largest diff. peak/hole / e Å^{-3}	0.25/-0.30

6. References

(1) Zou, L.; Braegelman, A. S.; Webber, M. J. Spatially Defined Drug Targeting by in Situ Host–Guest Chemistry in a Living Animal. *ACS Cent. Sci.* **2019**, *5* (6), 1035–1043. https://doi.org/10.1021/acscentsci.9b00195.

(2) Bouchene, R.; Daran, J.; Bouacida, S.; Manoury, E. Synthesis and Reactivity of Ferrocenyl-Substituted Allylamine Derivatives. *Eur. J. Inorg. Chem.* **2017**, *2017* (2), 340–350. https://doi.org/10.1002/ejic.201600860.

(3) Hellmuth, T.; Rieckhoff, S.; Weiss, M.; Dorst, K.; Frey, W.; Peters, R. Cooperative Bimetallic Asymmetric Catalysis: Comparison of a Planar Chiral Ruthenocene Bis-Palladacycle to the Corresponding Ferrocene. *ACS Catal.* **2014**, *4* (6), 1850–1858. https://doi.org/10.1021/cs500393x.

(4) Beagley, P.; Blackie, M. A. L.; Chibale, K.; Clarkson, C.; Moss, J. R.; Smith, P. J. Synthesis and Antimalarial Activity in Vitro of New Ruthenocene–Chloroquine Analogues. *J. Chem. Soc., Dalton Trans.* **2002**, No. 23, 4426–4433. https://doi.org/10.1039/B205432A.

(5) Andersen, C. B.; Wan, Y.; Chang, J. W.; Riggs, B.; Lee, C.; Liu, Y.; Sessa, F.; Villa, F.; Kwiatkowski, N.; Suzuki, M.; Nallan, L.; Heald, R.; Musacchio, A.; Gray, N. S. Discovery of Selective Aminothiazole Aurora Kinase Inhibitors. *ACS Chem. Biol.* **2008**, *3* (3), 180–192. https://doi.org/10.1021/cb700200w.

(6) Cai, J.; Chen, X.; You, H.; Li, X.; Ji, M. Design, Synthesis and Activity Evaluation of Hedgehog Inhibitor Itraconazole Derivatives in A549 Cells. *Bioorg. & Med. Chem. Lett.* **2022**, *76*, 129011. https://doi.org/10.1016/j.bmcl.2022.129011.

(7) Pace, J. R.; DeBerardinis, A. M.; Sail, V.; Tacheva-Grigorova, S. K.; Chan, K. A.; Tran, R.; Raccuia, D. S.; Wechsler-Reya, R. J.; Hadden, M. K. Repurposing the Clinically Efficacious Antifungal Agent Itraconazole as an Anticancer Chemotherapeutic. *J. Med. Chem.* **2016**, *59* (8), 3635–3649. https://doi.org/10.1021/acs.jmedchem.5b01718.

(8) Lombardo, F. C.; Pasche, V.; Panic, G.; Endriss, Y.; Keiser, J., Life cycle maintenance and drug-sensitivity assays for early drug discovery in Schistosoma mansoni. *Nature protocols* **2019,** *14* (2), 461-481. DOI: 10.1038/s41596-018-0101-y.

(9) Mäser, P.; Grether-Bühler, Y.; Kaminsky, R.; Brun, R., An anti-contamination cocktail for the in vitro isolation and cultivation of parasitic protozoa. *Parasitology Research* **2002,** *88* (2), 172-174. DOI: 10.1007/s00436-001-0511-5.

(10) Buchter, V.; Schneeberger, P. H. H.; Keiser, J., Validation of a human-serum-based in vitro growth method for drug screening on juvenile development stages of Schistosoma mansoni. *PLoS Negl Trop Dis* **2021,** *15* (3), e0009313. DOI: 10.1371/journal.pntd.0009313.

(11) Keiser, J.; Haberli, C., Evaluation of Commercially Available Anthelminthics in Laboratory Models of Human Intestinal Nematode Infections. *ACS Infect Dis* **2021,** *7* (5), 1177-1185. DOI: 10.1021/acsinfecdis.0c00719.

(12) Probst, A.; Chisanga, K.; Dziwornu, G. A.; Haeberli, C.; Keiser, J.; Chibale, K., Expanding the Activity Profile of Pyrido[1,2-a]benzimidazoles: Synthesis and Evaluation of Novel N(1)-1-Phenylethanamine Derivatives against Schistosoma mansoni. *ACS Infect Dis* **2021,** *7* (5), 1032-1043. DOI: 10.1021/acsinfecdis.0c00278.

(13) Page, B.; Page, M.; Noel, C., A NEW FLUOROMETRIC ASSAY FOR CYTOTOXICITY MEASUREMENTS IN-VITRO. *Int J Oncol* **1993,** *3* (3), 473-476. DOI: 10.3892/ijo.3.3.473.

(14) Ansar Ahmed, S.; Gogal, R. M.; Walsh, J. E., A new rapid and simple non-radioactive assay to monitor and determine the proliferation of lymphocytes: an alternative to [3H]thymidine

incorporation assay. *Journal of Immunological Methods* **1994,** *170* (2), 211-224. DOI: https://doi.org/10.1016/0022-1759(94)90396-4.

(15) Huber, W.; Koella, J. C., A comparison of three methods of estimating EC50 in studies of drug resistance of malaria parasites. *Acta Trop* **1993,** *55* (4), 257-61. DOI: 10.1016/0001-706x(93)90083-n.

(16) Clark, R. C.; Reid, J. S. The Analytical Calculation of Absorption in Multifaceted Crystals. *Acta Crystallogr. A* **1995**, *51* (6), 887–897. https://doi.org/10.1107/S0108767395007367.

(17) Rigaku Oxford Diffraction Ltd. CrysAlisPro(Version 1.171.43.97a). *Yarnton* **2023**, *Oxfordshire*, England.

(18) Dolomanov, O. V.; Bourhis, L. J.; Gildea, R. J.; Howard, J. A. K.; Puschmann, H. *OLEX2* : A Complete Structure Solution, Refinement and Analysis Program. *J. Appl. Crystallogr.* **2009**, *42* (2), 339–341. https://doi.org/10.1107/S0021889808042726.

(19) Sheldrick, G. M. *SHELXT* – Integrated Space-Group and Crystal-Structure Determination. *Acta Crystallogr. Sect. Found. Adv.* **2015**, *71* (1), 3–8. https://doi.org/10.1107/S2053273314026370.

(20) Sheldrick, G. M. Crystal Structure Refinement with *SHELXL*. *Acta Crystallogr. Sect. C Struct. Chem.* **2015**, *71* (1), 3–8. https://doi.org/10.1107/S2053229614024218.

(21) Spek, A. L. Structure Validation in Chemical Crystallography. *Acta Crystallogr. D Biol. Crystallogr.* **2009**, *65* (2), 148–155. https://doi.org/10.1107/S090744490804362
